# Suppression of GRK2 expression reduces endothelial dysfunction by restoring glucose homeostasis

**DOI:** 10.1038/s41598-017-08998-5

**Published:** 2017-08-16

**Authors:** Kumiko Taguchi, Mari Hida, Mami Hasegawa, Haruka Narimatsu, Takayuki Matsumoto, Tsuneo Kobayashi

**Affiliations:** Department of Physiology and Morphology, Institute of Medicinal Chemistry, Hoshi University, Shinagawa-ku, Tokyo 142-8501 Japan

## Abstract

Despite the associations between diabetic complications and vascular endothelial dysfunction, a direct therapeutic method targeting endothelial dysfunction remains poorly characterized. We have previously shown that chemical inhibition of G-protein-coupled receptor kinase 2 (GRK2) slightly enhances insulin sensitivity and reduces endothelial dysfunction in type 2 diabetic mice. In this study, we identified GRK2 as a novel therapeutic target of diabetic endothelial dysfunction and investigated the effect on diabetic endothelial dysfunction through the systemic administration of GRK2 siRNA using a hydrodynamic-based procedure, resulting in suppression of increased GRK2 protein levels in the liver. Suppressed GRK2 levels in the liver markedly improved glucose homeostasis, as well as improved the impaired endothelial Akt/eNOS-dependent signal activation (insulin-stimulated phosphorylation of Akt and eNOS) and vascular responses (clonidine-induced and insulin-induced endothelial-dependent relaxation response and phenylephrine-induced contractile response) in type 2 diabetic aortas. Interestingly, insulin-stimulated Akt/eNOS signaling was increased only by normalizing the glucose concentration in human umbilical vein endothelial cells (HUVECs) with GRK2 overexpression, suggesting of an important role of hepatic GRK2. Our results clarified the relationship among hepatic GRK2, glucose homeostasis, and vascular endothelial function. Liver-targeting GRK2 siRNA delivery represents a novel therapeutic tool to restore glucose homeostasis and reduce endothelial dysfunction.

## Introduction

Individuals with long-term type 2 diabetes mellitus (T2DM) and chronically poor metabolic control manifest vascular diseases. Most classical and novel cardiovascular disease (CVD)-associated risk factors, including T2DM combine to impair endothelial function^1–3^. Endothelial dysfunction is a marker for subclinical disease, an independent predictor of adverse CVD events, and a potential target for medical intervention^2, 4–6^.

G protein-coupled receptor kinase 2 (GRK2), a family of seven serine/threonine protein kinases that specifically recognize and phosphorylate agonist-activated G protein-coupled receptors (GPCRs) is emerging as a key integrative node in many signaling pathways that directly interacts with and/or phosphorylates non-GPCR components of transduction cascades^7–9^. Other laboratories and ours have shown that GRK2 inhibitors improve glucose homeostasis and the insulin response^10–13^. GRK2 is highly expressed in many tissues, including blood mononuclear cells, human visceral adipocytes, white adipose tissue, and muscle and liver tissue of metabolic syndrome patients and model animals^14^. Moreover, increased levels of GRK2 can decrease insulin signaling in several cell types and tissues^14, 15^.

Insulin suppresses hepatic glucose production. In the liver, a key organ in metabolic regulation, Vila-Bedmar *et al*.^16^ have shown that suppression of GRK2 in fatty liver protected against hepatic insulin resistance. Furthermore, Insulin stimulates endothelial nitric oxide synthase (eNOS) activity by Akt activation, leading to eNOS phosphorylation at Ser1177, NO production and vasorelaxation^17–19^. Evidence from our, and several other, experimental models of insulin resistance and T2DM has revealed impaired insulin-mediated Akt/eNOS-dependent signaling in the vasculature^20–24^. We have recently discovered that GRK2 promotes endothelial dysfunction via suppression of the insulin-stimulated Akt/eNOS/NO signaling pathway^23, 24^. However, it remains unknown whether altering glucose tolerance and insulin resistance regulates insulin-mediated Akt signaling-related GRK2 in T2DM models.

In this study, we treated with systemic GRK2 small interfering RNA (siRNA) for suppression of GRK2. Several studies describing gene delivery have appeared. We had previously focused on the hydrodynamic injection method, in which a large volume of naked nucleic acids including plasmid DNA and siRNA is rapidly injected into the tail vein of mice, resulting in effective nuclear transgene expression in hepatocytes^25–28^. In our previous study, western blot analysis confirmed that adiponectin gene injection achieved delivery of naked DNA to the liver^27^. However, to our knowledge, hydrodynamic injection of GRK2 siRNA has not been reported.

Our overall hypothesis was that GRK2 siRNA arrives at the hepatocytes using the hydrodynamic injection method, and hepatic GRK2 acts as an extensive metabolic regulator, owing to its ability to directly target insulin sensitivity, glucose metabolism, and vascular function. To propose GRK2 as a potential drug target in T2DM, it would be necessary to target organs for GRK2 and show that suppression of those GRK2 can reverse chronic endothelial dysfunction by restoring glucose homeostasis and insulin sensitivity. In the present study, we investigated the relationships among the expression profile of GRK2 in the liver, the direct effect of insulin sensitivity, and Akt/eNOS-mediated vascular endothelial function. Specifically, we attempted to determine whether the GRK2 siRNA has an effect on restoration of glucose homeostasis, which culminate in impairment of the endothelial-dependent vascular response by the activation of the Akt/eNOS signaling pathway.

## Results

### GRK2 expression in multiple tissues

**Figure 1:**
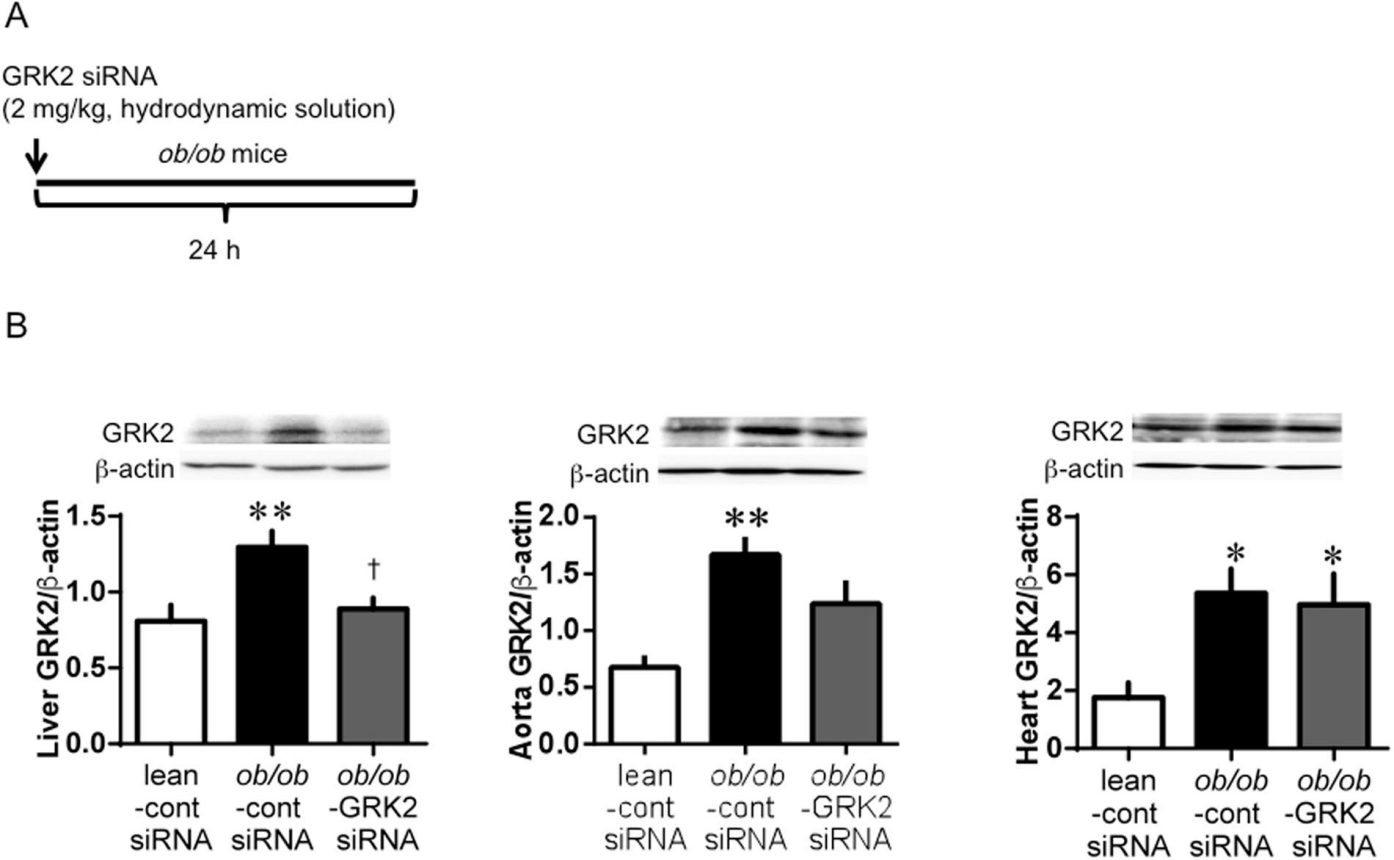
GRK2 is reduced by GRK2 siRNA delivery in liver. (**A**) Experimental protocol for *ob/ob* mice: *ob/ob* mice or lean (control) mice were treated with GRK2 siRNA or control siRNA by hydrodynamic delivery as indicated (intravenously, 2 mg/kg). (**B**) Western blot analysis of GRK2 in the liver, aorta and heart, respectively. Top is the representative Western blots for GRK2 protein, and bottom is the total GRK2 expression. Ratios were calculated for the optical density of GRK2 over that of β-actin. Values are mean ± SE; *n* = 6. ^*^ *p* < 0.05, ^**^ *p* < 0.01 vs. control siRNA-transfected lean mice (lean-cont siRNA); ^†^ *p* < 0.05 vs. control siRNA-transfected *ob/ob* mice (*ob/ob*-cont siRNA) by one-way analysis of variance.

To obtain suppressed GRK2 expression, mice received a hydrodynamic injection of GRK2 siRNA (Fig. 1A and Supplementary Fig. 1A). Experimental T2DM (nicotinamide- streptozotocin (STZ) induced diabetic) mice showed markedly increased GRK2 protein levels from those in the control mice in the liver, aorta, and heart (Supplementary Fig. 1B). Hydrodynamic injection of GRK2 siRNA decreased the GRK2 protein levels, which changed depending on the GRK2 siRNA dose in experimental T2DM mice. At 24 h after GRK2 siRNA injection, a maximum of 50% reduction in the GRK2 levels from those in the no-injection experimental T2DM mice was achieved with the 2 mg/kg dose level (Supplementary Fig. 1B and C). Accordingly, the effective dose of GRK2 siRNA in this study was determined to be 2 mg/kg. In genetic T2DM (*ob/ob*) mice, hydrodynamic injection of control siRNA increased the GRK2 levels from those in the lean mice in the liver, aorta and heart (Fig. 1B). By contrast, control siRNA injection decreased the GRK2 protein levels in the pancreas of *ob/ob* mice and did not change these levels in the skeletal muscle or lungs of *ob/ob* mice (Supplementary Fig. 2). Hydrodynamic injection of GRK2 siRNA decreased the GRK2 protein levels in the liver but not in other organs of the *ob/ob* mice, confirming the liver specificity of the procedure.

### Body weight and basal glycemic levels

**Figure 2:**
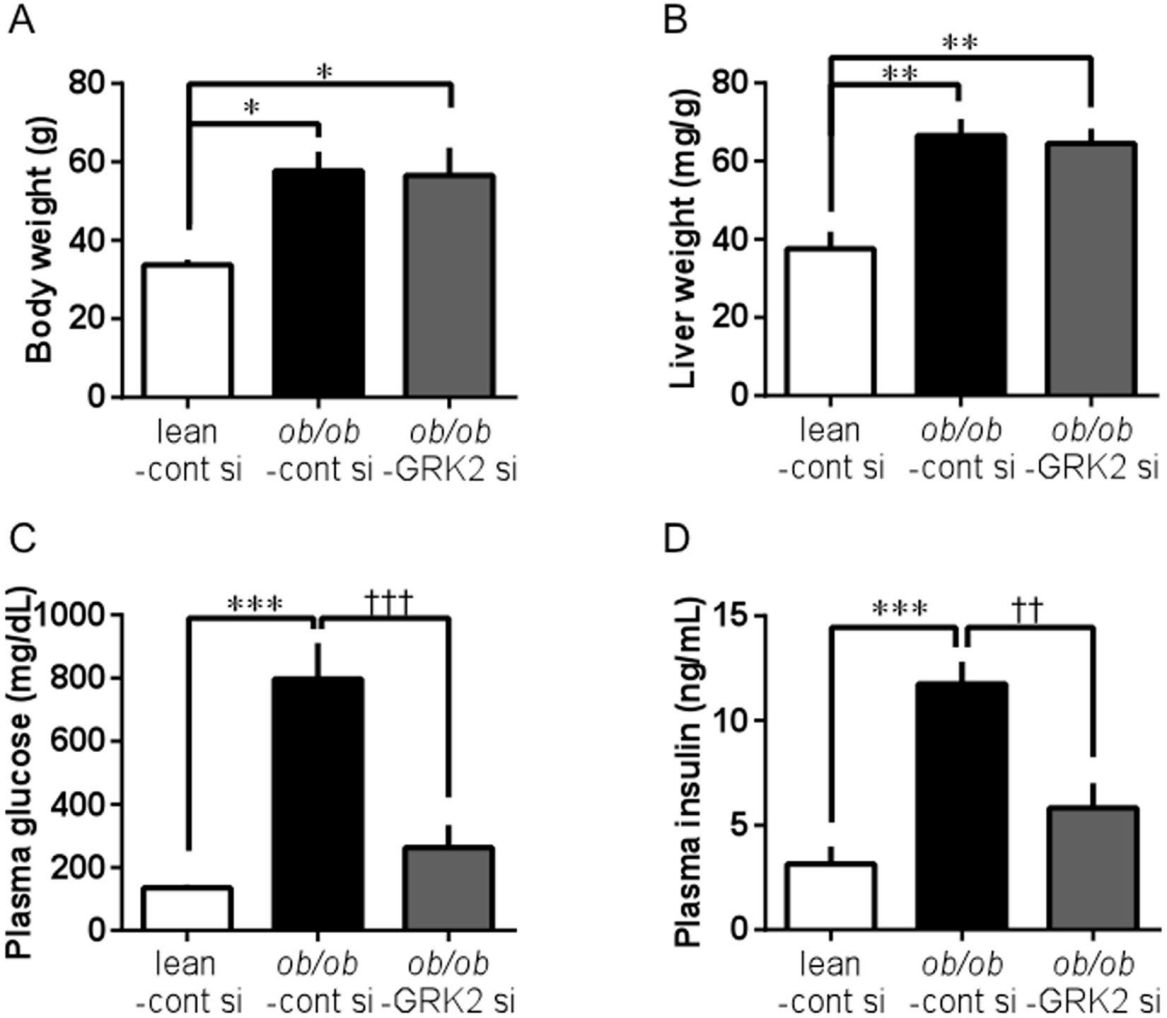
Plasma levels of glucose and insulin are improved in *ob/ob* mice by suppression of hepatic GRK2. (**A**) Body weight. (**B**) Liver weight. (**C**) Non-fasting plasma glucose levels. (**D**) Non-fasting plasma insulin levels. Values are mean ± SE; *n* = 6. ^*^ *p* < 0.05, ^**^ *p* < 0.01, ^***^ *p* < 0.001 vs. control siRNA-transfected lean mice (lean-cont si); ^††^ *p* < 0.01, ^†††^ *p* < 0.001 vs. control siRNA-transfected *ob/ob* mice (*ob/ob*-cont si) by one-way analysis of variance.

*Ob/ob* mice transfected with GRK2 siRNA and control siRNA showed marked obesity (Fig. 2A). No significant difference in liver weight was observed between the *ob/ob* mice transfected with GRK2 siRNA and those transfected with control siRNA (Fig. 2B). The non-fasting glucose and insulin levels of the control siRNA-transfected *ob/ob* mice were significantly higher than those of the control siRNA-transfected lean mice (Fig. 2C and D). These levels in the GRK2 siRNA-transfected *ob/ob* mice were significantly reduced from those of the control siRNA-transfected *ob/ob* mice. Importantly, the reductions in glucose and insulin levels were due to GRK2 siRNA-induced suppression in hepatic GRK2 function, given that there were no changes in these levels in the GRK2-inhibitor-treated *ob/ob* mice (Supplementary Fig. 3).

### GRK2 siRNA improves metabolic parameters in *ob/ob* mice

**Figure 3:**
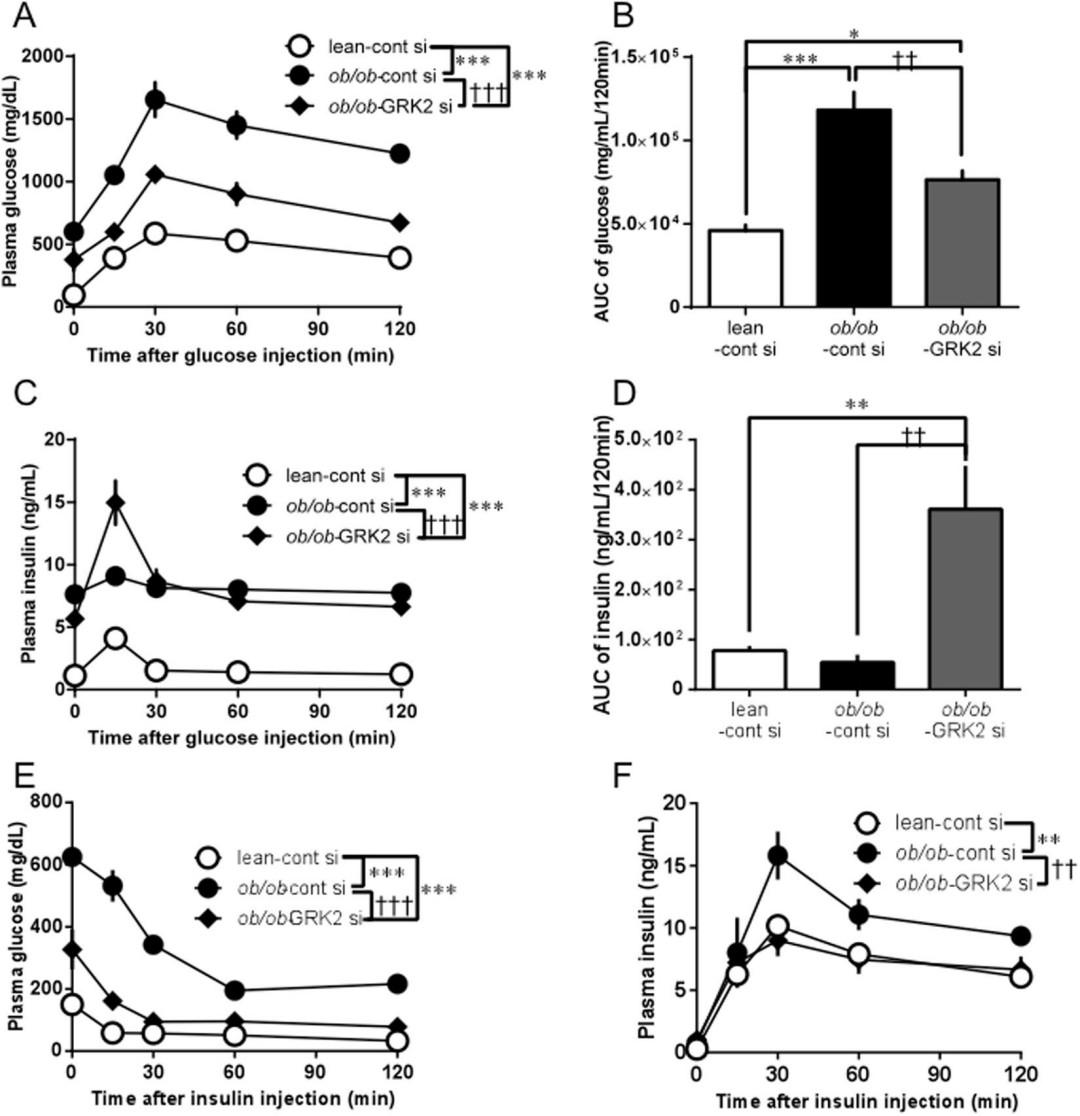
Suppression of hepatic GRK2 restores glucose homeostasis and insulin sensitivity in *ob/ob* mice. (**A–D**) Plasma levels of glucose (**A,B**) and insulin (**C,D**) were determined during a IGTT (glucose 2.0 g/kg intraperitoneally [i.p.]) performed 24 h after GRK2 siRNA injection. The AUC of glucose (**B**) and insulin (**D**). (**E,F**) Plasma levels of glucose (**E**) and insulin (**F**) were determined during an IITT (insulin 0.75 U/kg i.p.) performed after 24 h of GRK2 siRNA injection. Values are mean ± SE; *n* = 6. ^*^ *p* < 0.05, ^**^ *p* < 0.01, ^***^ *p* < 0.001 vs. control siRNA-transfected lean mice (lean-cont si); ^††^ *p* < 0.01, ^†††^ *p* < 0.001 vs. control siRNA-transfected *ob/ob* mice (*ob/ob*-cont si) by repeated measures two-factor ANOVA test followed by Bonferroni correction for multiple comparisons (**A,C,E,F**) or one-way analysis of variance (**B,D**).

To further investigate the effect of GRK2 siRNA on carbohydrate metabolism, we performed glucose tolerance tests (GTTs) and insulin tolerance tests (ITTs) 24 h after GRK2 siRNA injection. Control siRNA-transfected *ob/ob* mice showed greater glucose intolerance than the control siRNA-transfected lean mice, and the GRK2 siRNA-transfected *ob/ob* mice showed an improvement in glucose clearance from that in the control siRNA-transfected *ob/ob* mice, as shown by the GTTs (Fig. 3A–D). Indeed, of all the groups, the control siRNA-transfected *ob/ob* mice yielded the largest areas under the curve (AUC) and the highest post-loading glucose peaks at 30 min (Supplementary Fig. 4A), and those AUC levels and glucose peaks of GRK2 siRNA-transfected *ob/ob* mice changed to those levels of the control siRNA-transfected lean mice. The corresponding post-loading insulin profiles showed that the GRK2 siRNA or control siRNA-transfected *ob/ob* mice had significantly higher basal insulin levels than the control siRNA-transfected lean mice did and that the GRK2 siRNA-transfected *ob/ob* mice showed significantly augmented insulin levels 15 min after glucose loading (Supplementary Fig. 4B).

An ITT showed that the control siRNA-transfected *ob/ob* mice were insulin-resistant, as indicated by their significantly lower glucose clearance from the bloodstream in response to administered insulin. In the GRK2 siRNA-transfected *ob/ob* mice, the glucose levels were closer to those of control siRNA-transfected lean mice than to those of the *ob/ob* mice (Fig. 3E), whereas the insulin levels of the GRK2 siRNA-transfected *ob/ob* mice were superimposable onto those of the control siRNA-transfected lean mice (Fig. 3F). In the GRK2 siRNA-transfected *ob/ob* mice, the hypoglycemic effects of insulin administration showed a trend similar to that in the control siRNA-transfected lean mice (Supplementary Fig. 4C).

### Suppression of GRK2 protein levels reduces impaired vascular function

**Figure 4:**
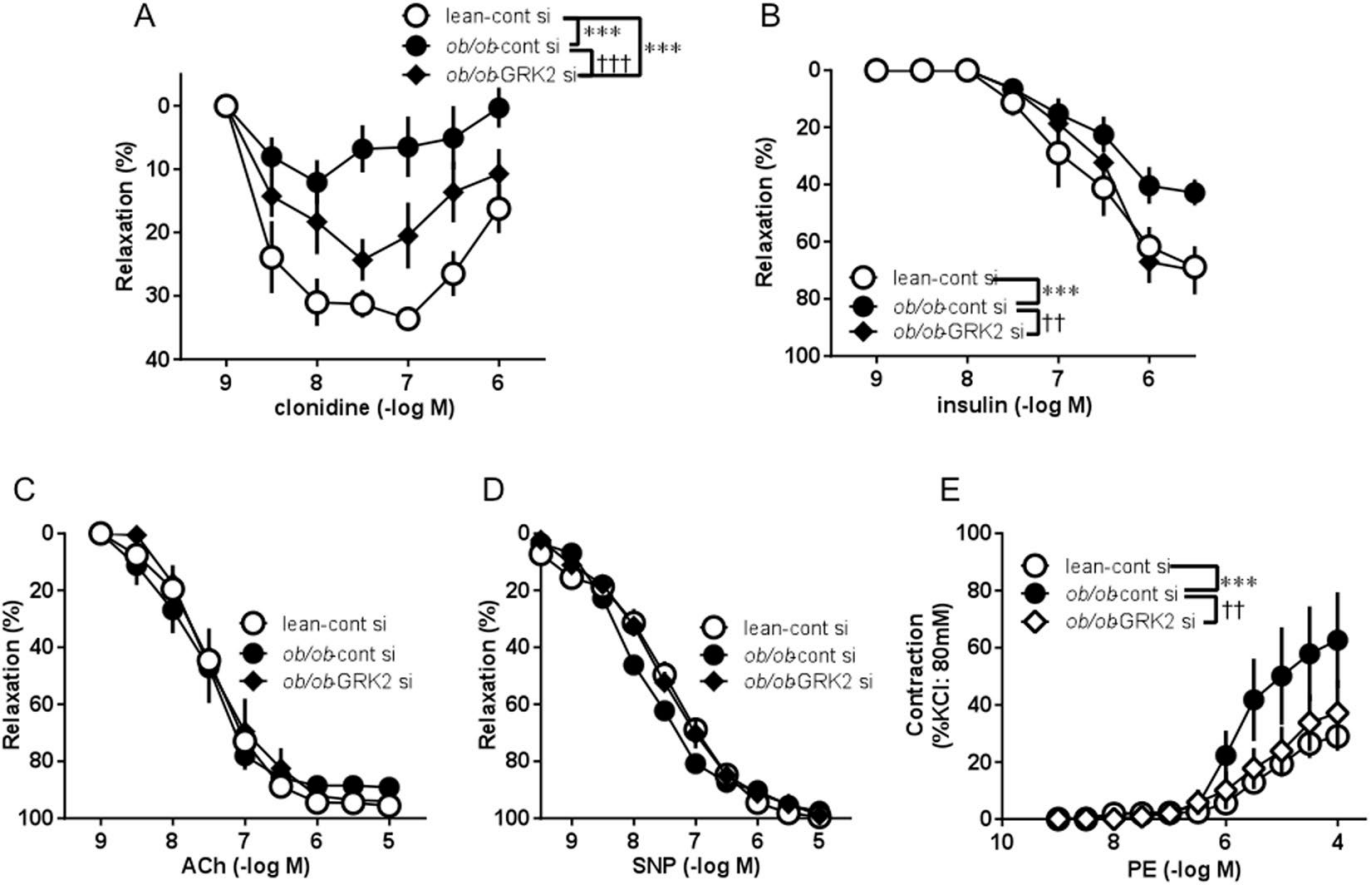
Suppression of hepatic GRK2 results in vascular function. Concentration response curves for clonidine (**A**), insulin (**B**), acetylcholine (ACh; **C**), sodium nitroprusside (SNP; **D**), and phenylephrine (PE; **E**) in aortas from control siRNA-transfected lean mice (lean-cont si), control siRNA-transfected *ob/ob* mice (*ob/ob*-cont si), and GRK2 siRNA-transfected *ob/ob* mice (*ob/ob*-GRK2 si). Values are mean ± SE; *n* = 4–5. ^***^ *p* < 0.001 vs. lean-cont si; ^††^ *p* < 0.01, ^†††^ *p* < 0.001 vs. *ob/ob*-cont si by repeated measures two-factor ANOVA test followed by Bonferroni correction for multiple comparisons.

In our previous studies, clonidine, α_2_-adrenergic receptor agonist, and insulin-induced endothelial relaxation responses were attenuated in diabetes^10, 22–24^. Clonidine-induced relaxation in the aortic rings of the control siRNA-transfected *ob/ob* mice was reduced from that in the control siRNA-transfected lean mice, but was improved by GRK2 siRNA transfection (Fig. 4A). Similarly, insulin-induced relaxation in the aortic rings of the control siRNA-transfected *ob/ob* mice was reduced from that in the control siRNA-transfected lean mice, but was restored by GRK2 siRNA transfection (Fig. 4B). As shown in Fig. 4C, GRK2 siRNA transfection had no effect on acetylcholine (ACh)-induced specific endothelium-dependent relaxation. Illustrating that GRK2 siRNA transfection specifically antagonized the activation of endothelial function in the mice aorta, the sodium nitroprusside (SNP)-induced endothelium-independent relaxation response is shown in Fig. 4D. GRK2 siRNA transfection had no effect on SNP-induced relaxation.

Vascular contraction in response to phenylephrine (PE), an α_1_-adrenergic receptor agonist, was augmented in the aortas of the control siRNA-transfected *ob/ob* mice but not in those of the GRK2 siRNA-transfected *ob/ob* mice (Fig. 4E).

To further test whether the improved vascular function in GRK2 siRNA-transfected *ob/ob* mice was due to changes in the hepatic GRK2 protein levels or due to changes in the vascular GRK2 protein levels, we measured the relaxation in response to clonidine and insulin, and contraction to PE, by treatment with a GRK2 specific-inhibitor by single injection (Supplementary Fig. 5). Interestingly, the GRK2 inhibitor had no effect on the clonidine-induced relaxation response or PE-induced contraction response in the aortas from *ob/ob* mice, but improved the insulin-induced relaxation response. Thus, these results suggested that GRK2 siRNA was effective against vascular dysfunction by the mechanism of GRK2 suppression in the liver, not by the direct mechanism of GRK2 suppression in the vascular.

### Suppression of hepatic GRK2 protein levels improves insulin signaling in aorta and liver

**Figure 5:**
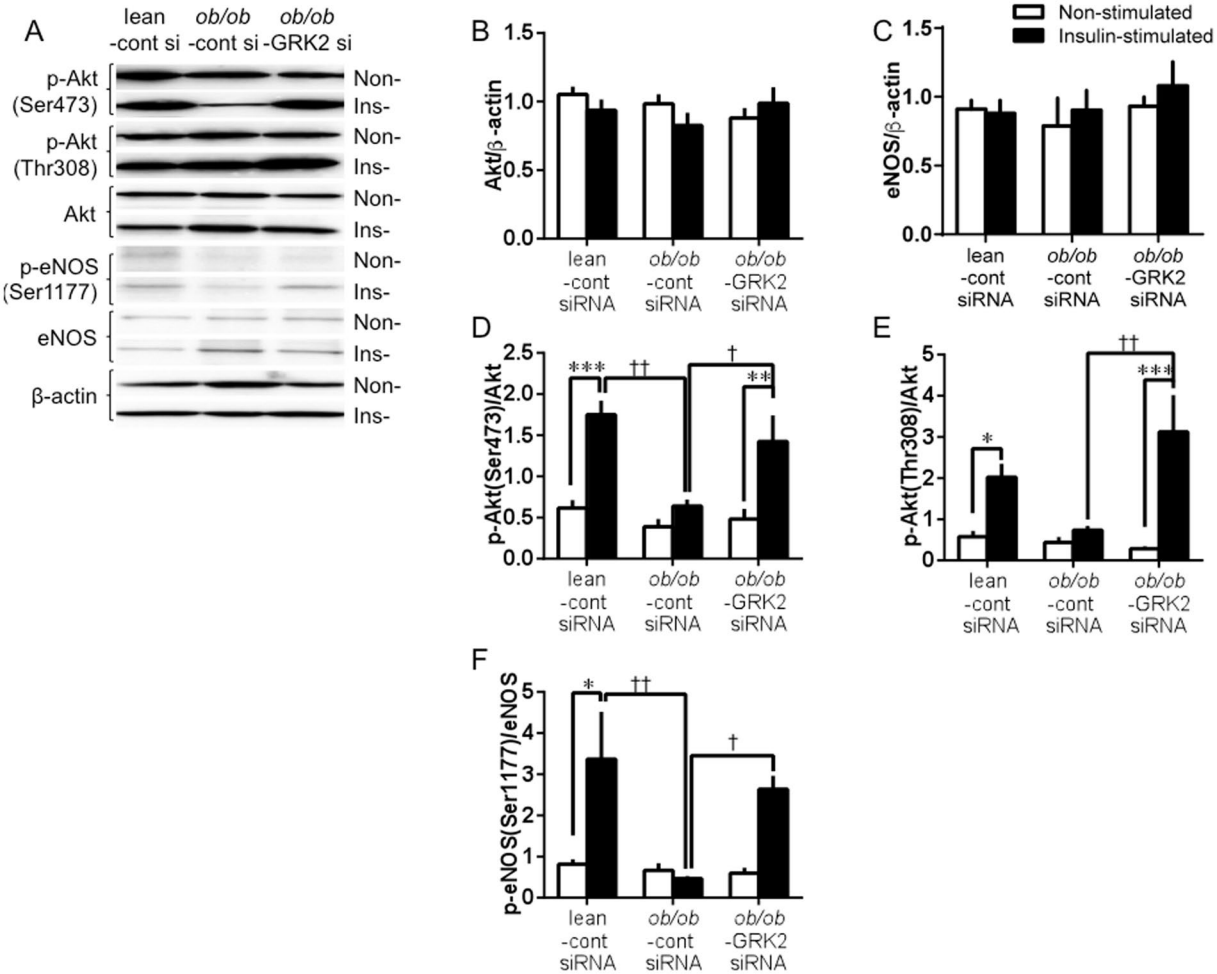
Effect of suppression of hepatic GRK2 on insulin signaling in *ob/ob* mice. (**A**) Representative western blot analysis of Akt phosphorylation (Ser473 and Thr308), total Akt, eNOS phosphorylation (Ser1177), total eNOS, and β-actin in lean and *ob/ob* aortas from mice unstimulated or stimulated with insulin (10^−6^ mol/L; 20 min). Basically, Insulin-stimulated and non-stimulated lanes were run on the same gel but were noncontiguous, and partly, were run on a separated gel but under the same conditions. (**B–F**) Quantification proteins are expressed as ratio of Akt to β-actin (**B**), ratio of eNOS to β-actin (**C**), ratio of Akt phosphorylation (at Ser473) to total Akt (p-Akt [Ser473]) (**D**), ratio of Akt phosphorylation (at Thr308) to total Akt (p-Akt[Thr308]) (**E**), and eNOS phosphorylation (at Ser1177) to total eNOS (p-eNOS [Ser1177]) (**F**). Phosphorylated proteins are normalized to total proteins. Total proteins are normalized to β-actin. Values are mean ± SE; *n* = 5–6. ^*^ *p* < 0.05, ^**^ *p* < 0.01, ^***^ *p* < 0.001 vs. non-stimulated; ^†^ *p* < 0.05, ^††^ *p* < 0.01 vs. control siRNA-transfected *ob/ob* mice (*ob/ob*-cont si) by one-way analysis of variance.

We examined the expression of Akt, eNOS, and their phosphorylated forms under insulin stimulation or non-stimulation in the aorta. The aortas in all groups exhibited similar levels of Akt and eNOS (Fig. 5A–C). Phospho-Akt and phospho-eNOS (Ser1177) were both significantly induced by insulin stimulation in the aortas from the control siRNA-transfected lean mice, and the expression levels of these phosphorylations were not altered by non-stimulation and insulin stimulation in the aortas from the control siRNA-transfected *ob/ob* mice (Fig. 5D–F). Interestingly, GRK2 siRNA transfection induced significant increases in phospho-Akt and phospho-eNOS (Ser1177) under insulin stimulation. In the GRK2 siRNA-transfected *ob/ob* mice, the phospho-eNOS (Thr495) levels, showing a tendency to increase under insulin stimulation in the control siRNA-transfected *ob/ob* mice, had returned to those of the control siRNA-transfected lean mice (Supplementary Fig. 6A). In agreement with this finding, the aortic NO production induced by insulin was greater in the GRK2 siRNA-transfected *ob/ob* mice than in the control siRNA-transfected *ob/ob* mice (Supplementary Fig. 6B). Moreover, the aortic mitogen-activated protein kinase (MAPK) activation (ERK1/2 and p38 MAPK) induced by insulin was elevated in the control siRNA-transfected *ob/ob* mice, but GRK2 transfection induced a significant decrease in MAPK activation in response to insulin stimulation (Supplementary Fig. 7).

To determine how GRK2 regulates glucose metabolism in T2DM, we examined the activation of AMP-activated protein kinase (AMPK) and Akt-eNOS under insulin stimulation in the liver. In the *ob/ob* mice, the total AMPK levels and AMPK activation were not changed by GRK2 siRNA transfection from those with control siRNA transfection (Supplementary Fig. 8). In the *ob/ob* mice, impaired insulin signaling (Akt/eNOS pathway) was restored by GRK2 siRNA transfection (Supplementary Fig. 9).

### Endothelial dysfunction returned to normal after treatment with normal glucose concentration in HUVECs

**Figure 6:**
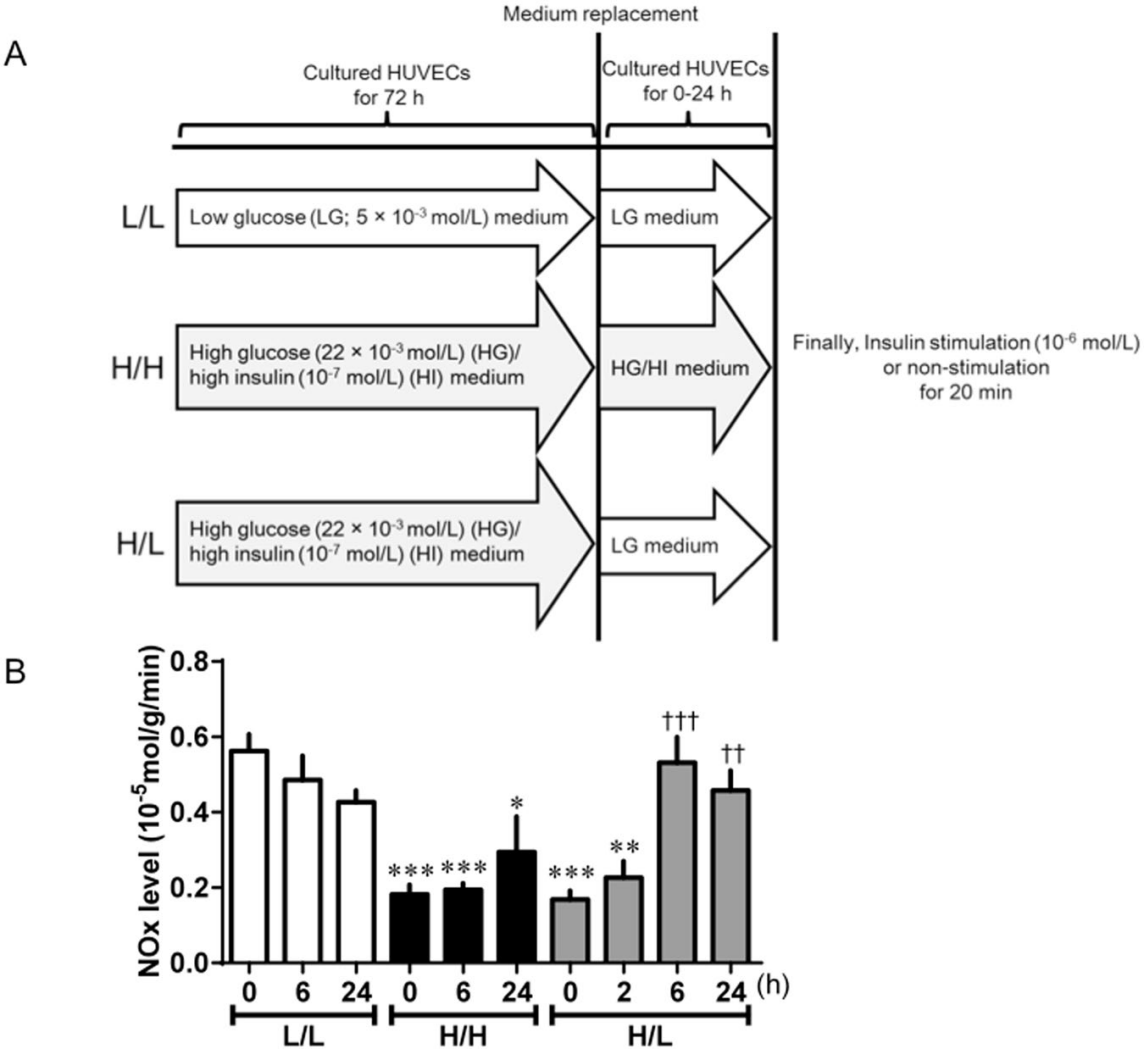
Normalization of glucose concentration improved impaired insulin-stimulated NO production in HUVECs induced GRK2 under high glucose (HG) and high insulin (HI). (**A**) Experimental protocol for HUVECs. L/L (Control) HUVECs were incubated in LG (5 × 10^−3^ mol/L) medium for 72 h, which was then replaced with the same medium. H/H (quasi-diabetic state) HUVECs were incubated in HG (22 × 10^−3^ mol/L)/HI (10^−7^ mol/L) medium for 72 h, which was then replaced with the same medium. H/L HUVECs were incubated in HG/HI medium for 72 h, which was then replaced with LG medium. (**B**) NO released from HUVECs was measured under insulin stimulation or non-stimulation. Data presented as insulin-stimulated NO production values after deduction of non-stimulated NO production values. HUVECs were treated with insulin (10^−6^ mol/L) for 20 min, 0, (2), 6 or 24 h after medium replacement. Values are mean ± SE; *n* = 6. ^*^ *p* < 0.05, ^**^ *p* < 0.01, ^***^ *p* < 0.001 vs. L/L (0 h); ^††^ *p* < 0.01, ^†††^ *p* < 0.001 vs. H/L (0 h) by one-way analysis of variance.

We sought to determine whether hepatic GRK2 siRNA transfection would improve endothelial dysfunction via GRK2 regulation of glucose metabolism and insulin sensitivity. We have previously shown that high glucose (HG)/high insulin (HI) conditions induce GRK2 in HUVECs^29^. We validated this method by culturing HUVECs under HG/HI conditions for 72 h. Western blot analysis showed that stimulation of HUVECs with high concentrations of glucose and insulin increased the levels of GRK2 (Supplementary Fig. 10A). Furthermore, hyperglycaemia induced endothelial dysfunction^30^. To determine whether the normalization of the glucose concentration can enhance NO production under insulin stimulation, we pretreated HUVECs with a HG/HI concentration for 72 h, replaced the medium with low glucose (LG) concentration and then stimulated the cells with 10^−6^ mol/L insulin for 20 min. Our results showed that under insulin stimulation, NO production and bioavailability from HUVECs treated and/or replaced with HG/HI remained decreased (Fig. 6 and Supplemental Fig. 10B). However, an increase in NO production was observed in the HG/HI medium treated/LG medium replaced-HUVECs after 6 h.

**Figure 7:**
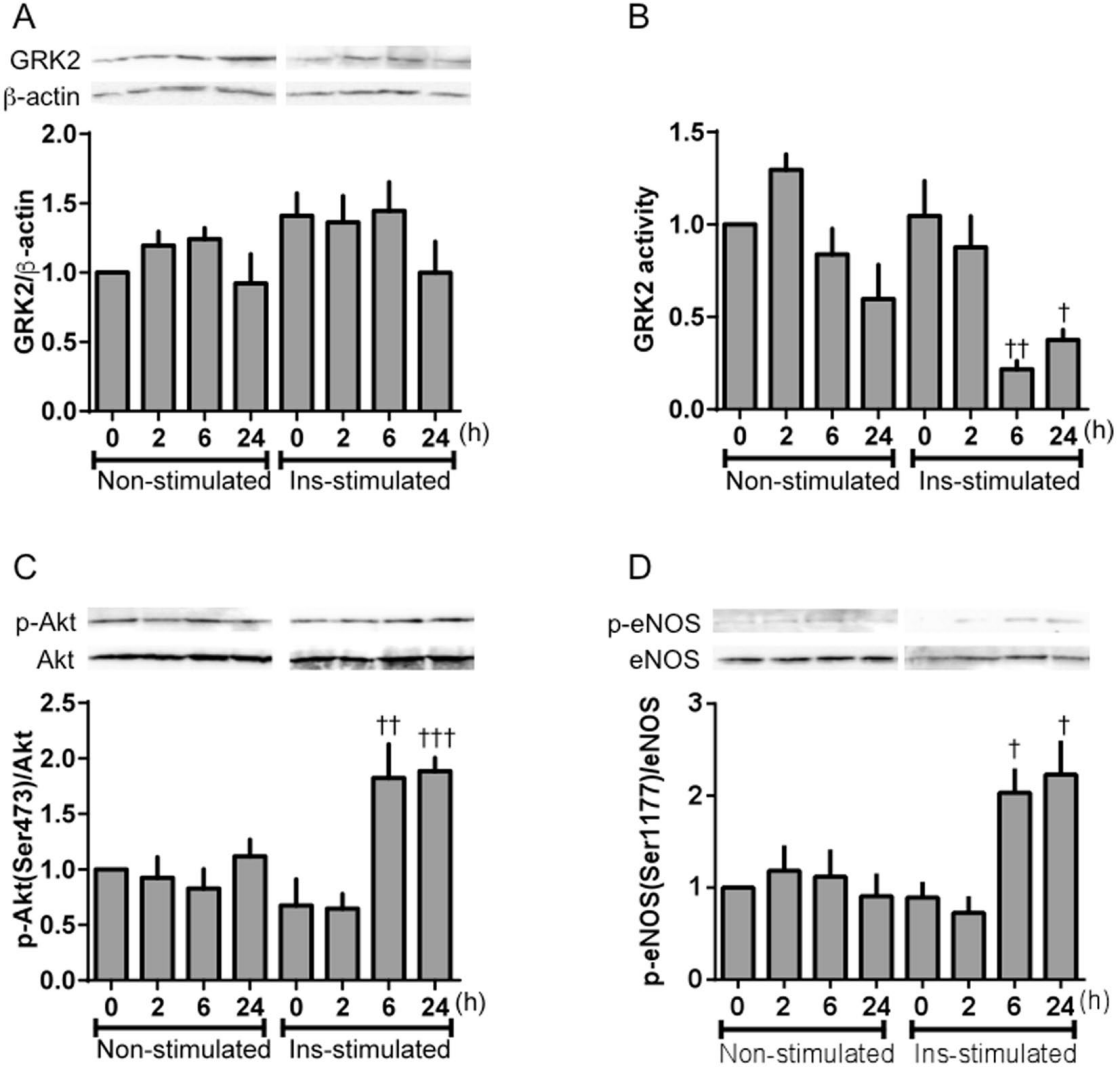
Normalization of glucose concentration improved impaired insulin signaling in HUVECs induced GRK2 under HG/HI conditions. HUVECs were incubated for 72 h in HG (22 × 10^−3^ mol/L)/HI (10^−7^ mol/L) medium, and then the medium was replaced with LG (5 × 10^−3^ mol/L) for 0-24 h. Finally, insulin (10^−6^ mol/L) was added for 20 min. GRK2 (**A**), phospho-Akt (**C**) and eNOS (**D**) were tested by western blotting assay. (**B**) Levels of GRK2 activity in HUVECs. Protein levels of phosphorylation were normalized to the levels of total protein. Basically, Insulin-stimulated and non-stimulated lanes were run on the same gel but were noncontiguous, and partly, were run on a separated gel but under the same conditions. Values are mean ± SE; *n* = 6. ^†^ *p* < 0.05, ^††^ *p* < 0.01, ^†††^ *p* < 0.001 vs. insulin-stimulated HUVECs incubated for 72 h in HG/HI medium and just behind the medium was replaced with LG (insulin-stimulated 0 h) by one-way analysis of variance.

**Figure 8:**
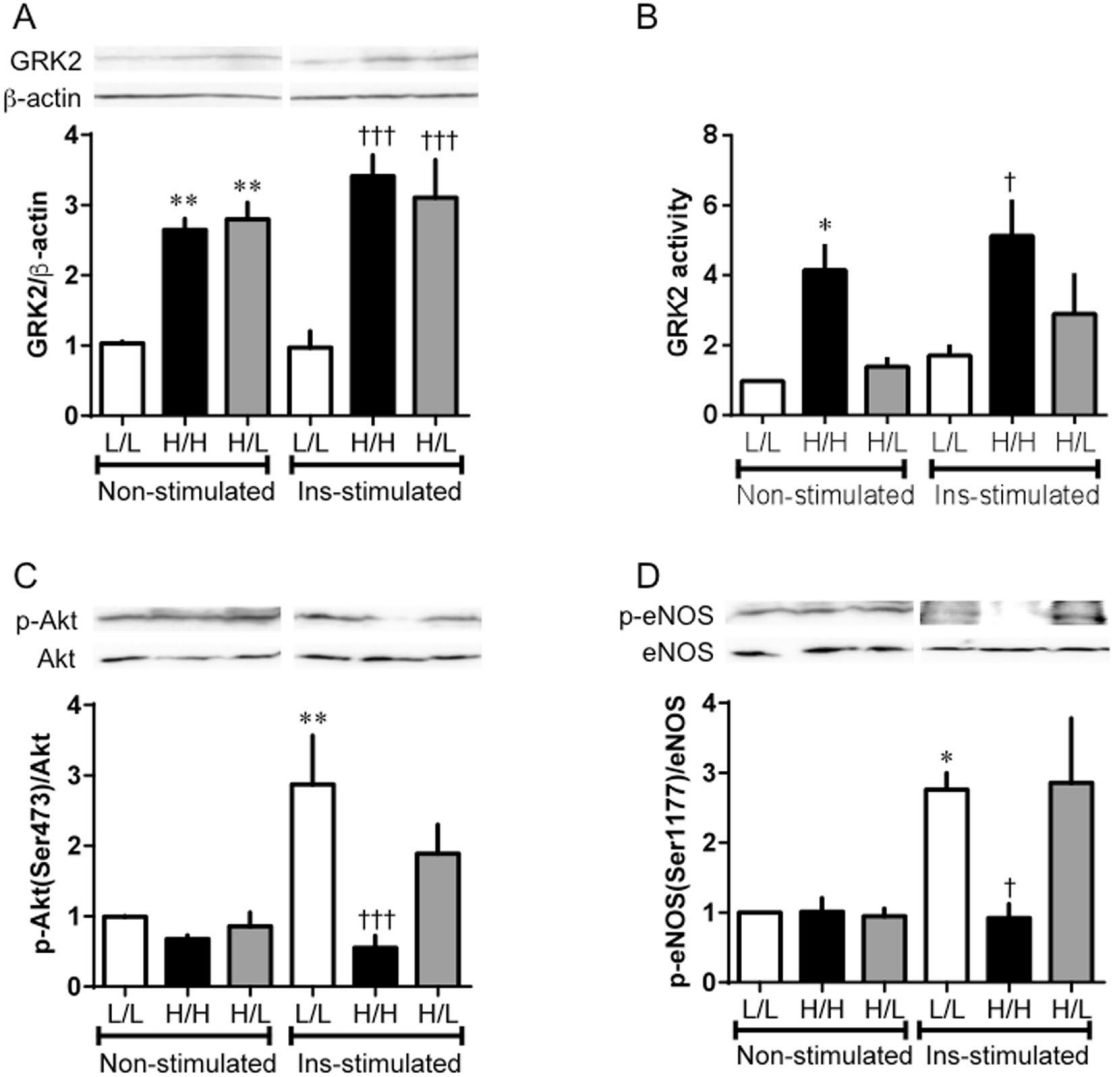
Acute normalization of glucose concentration improved impaired insulin signaling in HUVECs induced GRK2 underHG/HI conditions. HUVECs were incubated for 72 h in LG (5 × 10^−3^ mol/L)- or HG (22 × 10^−3^ mol/L)/HI (10^−7^ mol/L) medium, and then the medium was replaced for 6 h. Finally, insulin (10^−6^ mol/L) was added for 20 min. Abbreviations are defined in Fig. 6. GRK2 (**A**), phospho-Akt (**C**) and eNOS (**D**) were tested by western blotting assay. (**B**) Levels of GRK2 activity in HUVECs. Phosphorylated proteins are normalized to total proteins. Basically, Insulin-stimulated and non-stimulated lanes were run on the same gel but were noncontiguous, and partly, were run on a separated gel but under the same conditions. Values are mean ± SE; *n* = 6. ^*^ *p* < 0.05, ^**^ *p* < 0.01 vs. L/L (non-stimulated); ^†^ *p* < 0.05, ^†††^ *p* < 0.001 vs. L/L (insulin-stimulated) by one-way analysis of variance.

When HUVECs were incubated in HG/HI medium for 72 h and then the medium was replaced with LG medium, the GRK2 levels did not change under insulin stimulation or non-stimulation for up to 24 h (Fig. 7A). However, replacement with LG medium decreased the insulin-stimulated GRK2 activity and increased the insulin-stimulated phosphorylation of Akt and eNOS after 6 h (Fig. 7B–D). To confirm these observations further, in another set of experiments, HUVECs were incubated in LG medium or HG/HI medium for 72 h and then placed in LG or HG/HI medium for 6 h (Fig. 8). Six hours after medium replacement with the HG/HI, HUVECs showed a significant increase in the GRK2 levels and activity, and a significant decrease in insulin-induced Akt phosphorylation from that in the LG-treated cells. At 6 h post-medium replacement with LG, there was a decrease in GRK2 activity, and a significant increase in insulin-induced eNOS phosphorylation, although the GRK2 levels did not change in the cells replaced between in HG/HI and in LG.

Furthermore, we performed additional experiments. We studied whether clonidine-stimulated Akt/eNOS signaling and NO production in HUVECs increased GRK2 expression (Supplementary Fig. 11). We have previously reported that clonidine (an α_2_-receptor agonist) activated the Akt/eNOS pathway and increased NO production^10, 23^. Furthermore, GRK2 activation down-regulates Akt/eNOS signalling/NO production, leading endothelial dysfunction^10, 23, 24, 29, 31^. So, using HUVEC-induced GRK2 expression (Supplementary Fig. 11A), we studied clonidine-induced GRK2 activity, NO production, and Akt and eNOS phosphorylations, to determine whether the inhibition of GRK2 is required in endothelium. HG/HI medium increased the GRK2 activity and the GRK2 activity was completely blocked by a GRK2 inhibitor (Supplementary Fig. 11B). Our results showed that under clonidine stimulation, NO production from HUVECs treated HG/HI decreased, but in the presence of a GRK2 inhibitor, an increase in NO production was observed in the HG/HI medium (Supplementary Fig. 11C). Moreover, the clonidine-stimulated Akt and eNOS phosphorylations were significantly lower in HUVEC-treated HG/HI, and these phosphorylation responses were greater by GRK2 inhibitor in HUVEC-treated HG/HI (Supplementary Fig. 11D,E).

## Discussion

**Figure 9:**
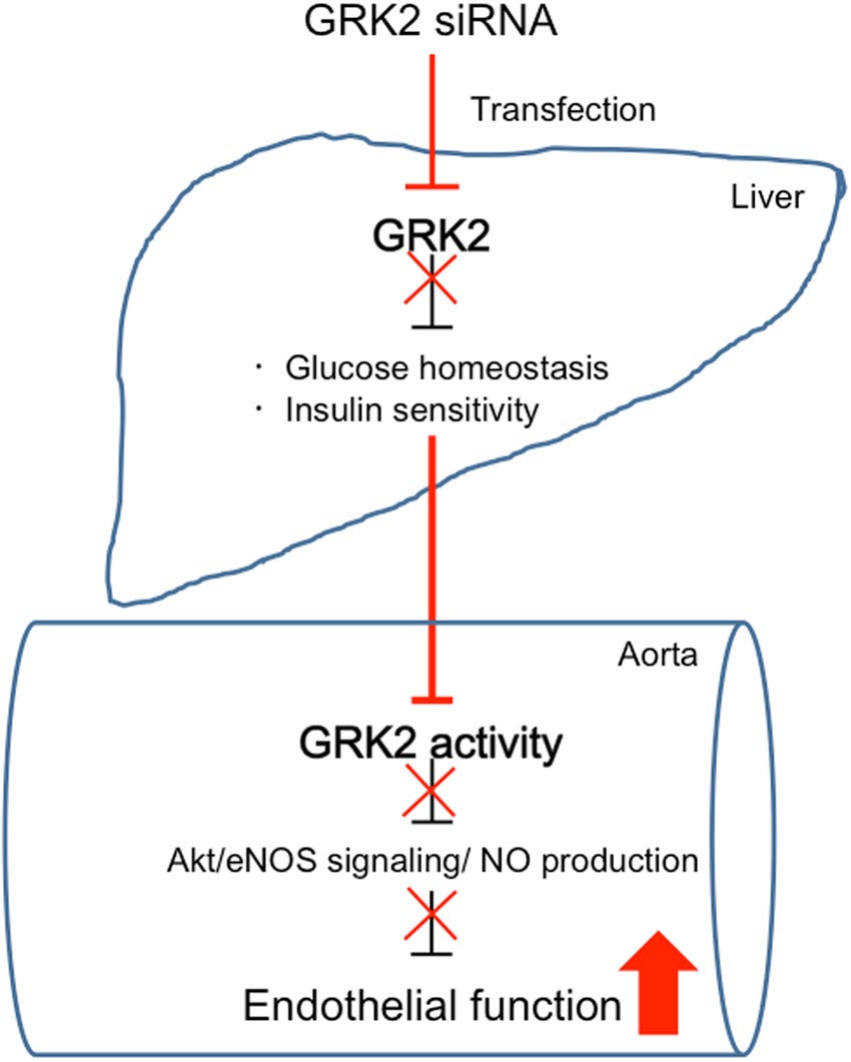
A model for interaction between liver and aorta based on GRK2 siRNA transfection in T2DM models. Hepatic GRK2 inhibition normalized the Akt/eNOS-dependent vascular response via improving glucose homeostasis in T2DM. Glucose homeostasis could constitute a network for replication between liver and vascular.

This study showed that hydrodynamic injection of GRK2 siRNA suppressed hepatic GRK2 protein levels and produced pronounced effects on the glucose metabolism and insulin sensitivity in *ob/ob* mice. We have reported previously that GRK2 is induced by high glucose and insulin levels in T2DM aortas and HUVEC cells, and that an increased GRK2 expression and activation inhibits Akt/eNOS signalling/NO production, leading to impaired endothelium-dependent vascular responses (endothelial dysfunction)^10, 23, 24, 29, 31^. The impaired endothelium-dependent vascular responses in *ob/ob* mice were improved in the aortas of GRK2 siRNA-transfected *ob/ob* mice. Following incubation of aortas from GRK2 siRNA-transfected *ob/ob* mice, Akt/eNOS phosphorylation was increased. Furthermore, increased insulin-stimulated Akt/eNOS phosphorylation was seen in replacement from HG/HI to LG medium in HUVECs. Our results demonstrate that the suppression of hepatic GRK2 dramatically improves systemic glucose homeostasis and insulin sensitivity and the rapidly normalised glucose homeostasis as well as of insulin sensitivity plays an important role in modulating endothelial function (Fig. 9).

Previous reports have indicated that GRK2 could act as an inhibitor of insulin action in cellular models and T2DM models. For example, increased GRK2 levels favored insulin resistance^32, 33^. Our group showed that GRK2 impaired the insulin-stimulated vascular response in the T2DM aortas^28^. However, the relationship between systemic insulin resistance and endothelial function in the aorta remains unclear. We investigated systemic glucose homeostasis and endothelium-dependent vascular responses in *ob/ob* mice transfected with GRK2 siRNA using adjusted hydrodynamic injection. Our results for the liver as well as the aorta and the heart showed that the GRK2 levels were elevated in the control siRNA-transfected *ob/ob* mice and that GRK2 siRNA transfection in *ob/ob* mice suppressed GRK2 expression only in the liver. Overall, these findings suggest that the GRK2 levels increased in various organs in T2DM and that GRK2 siRNA, by using adjusted hydrodynamic injection, entered the veins, was drawn into the liver. In view of these findings, we propose that GRK2 siRNA transfer positively regulated the liver, or at least did not directly regulate the aorta.

Previous studies have suggested that the activation of Akt/eNOS and endothelium-dependent relaxation induced by clonidine or insulin decreases in T2DM and that GRK2 influences endothelial function via the Akt/eNOS-dependent pathway^10, 22–24, 31^. Furthermore, in this study, increased endothelium-dependent relaxations in response to clonidine and insulin were shown in GRK2 siRNA-transfected *ob/ob* mice. The activation of Akt induced by clonidine or insulin can directly phosphorylate eNOS at Ser1177 and activate the enzyme, leading to NO production^22, 34^. In line with these previous reports, our data clearly show that GRK2 siRNA transfection increased Akt phosphorylations (Ser473 and Thr308) and eNOS phosphorylation (Ser1177), although control siRNA transfection decreased these phosphorylations in response to insulin stimulation in *ob/ob* mice. Furthermore, we observed that the increased PE-induced vasocontraction was restored in GRK2 siRNA-transfected *ob/ob* mice. This observation suggests that the absence of an effect of GRK2 on the PE response in aortas is associated with a lower decrease in NO, given that GRK2 reduces NO availability in the aorta^10, 23, 24^. However, a GRK2- inhibitor did not affect the contraction induced by PE, as shown in previous^10^ and supplemental data. This finding suggests that suppression of GRK2 in the liver has a protective regulation of the Akt/eNOS/NO production pathway in the aorta, given that GRK2 siRNA-transfected *ob/ob* mice did not exhibit elevated MAPK activation nor decreased NO bioavailability. GRK2 could be regulating vasodilator receptors preferentially to vasoconstrictor receptors^35, 36^.

In the present study using *ob/ob* mice, GRK2 siRNA transfection led to reduced plasma levels of glucose and insulin, although GRK2-inhibitor injection had no significant effects. Hepatic GRK2 deficiency resulted in improving glucose tolerance and enhancing insulin sensitivity. This finding suggests that GRK2 siRNA transfection has a more profound effect in mice with already altered insulin sensitivity and that GRK2 is a key modulator of insulin sensitivity *in vivo*. In fact, in T2DM models, GRK2 inhibition results in improved glucose homeostasis^10, 11, 33^. Glucose metabolism is regulated mainly by two pathways: the insulin signaling and the AMPK pathways^37^. In the present data, GRK2 siRNA transfection specifically caused a somewhat expected overt improvement of glucose homeostasis by regulation of hepatic insulin signaling. It remains to be established whether inhibition of GRK2 in the liver reduces endothelial dysfunction by restoration of glucose homeostasis and insulin sensitivity or vice versa.

To prove at cellular level, we investigated the interaction between glucose homeostasis and endothelial function in HUVECs. In a previous study, GRK2 levels and activities were increased when HUVECs were cultured under conditions of HG with HI levels^29^. Consistently, chronic high insulin levels have recently been shown to increase GRK2 levels and activities^31, 33, 37, 38^, findings in agreement with the upregulation of GRK2 observed in the aorta after insulin treatment. Thus, it is tempting to suggest that the hyperinsulinemia and hyperglycemia associated with insulin-resistant conditions, and clinically associated with T2DM, trigger the observed upregulation of GRK2 levels and activities under such conditions. As expected^29^, in the present study, HG/HI stimulation induced GRK2 levels and activities in HUVECs. Accordingly, we used the GRK2-induced HUVECs for subsequent experiments. Consequently, insulin-stimulated Akt/eNOS phosphorylations and NO production were increased by the replacement of HG/HI with LG medium for only 6 h, although GRK2 remained upregulated in HUVECs. Substantial clinical and experimental evidence has suggested that both hyperglycemia and hyperinsulinemia contribute to endothelial dysfunction^39–41^. Our results show that rapid improvement of glucose concentration leads directly to greater insulin-stimulated Akt/eNOS signal activation and higher NO production levels in endothelial cells, and cures the endothelial dysfunction. We have previously reported that both GRK2 expression and activity were increased in diabetic aortas under non-stimulation^10, 23, 24, 31^. Activated GRK2 evoked endothelial dysfunction by causing damaged Akt/eNOS signalling in aortas from diabetic mice^10, 23, 24, 31^. Furthermore, the results depicted in Supplementary Fig. 11 suggest that 1) the GRK2-induced endothelial cells had high GRK2 activity, 2) the high activity of GRK2 controls Akt/eNOS signalling and NO production, and 3) these endothelial functions are inhibited by a GRK2 inhibitor. Our findings suggest a direct relationship between GRK2 and vascular endothelial cells, and reveal that a rapid improvement in glucose concentrations directly leads to enhanced insulin-stimulated Akt/eNOS signalling and NO production in HUVECs. Furthermore, we observed that increased GRK2 activity was reduced only by replacement with LG medium, although increased GRK2 levels were maintained in HUVECs. Furthermore, in HUVECs, Akt/eNOS signalling was normalised only by a decrease in GRK2 activity. Given that GRK2 siRNA can normalize impaired glucose metabolism and insulin sensitivity in the liver, it may well constitute a protective factor against vascular endothelial dysfunction.

In conclusion, our study using *ob/ob* mice has shown that inhibition of hepatic GRK2 expression is sufficient to improve glucose homeostasis and insulin sensitivity, which eventually improves endothelial dysfunction in T2DM. In agreement with these observations, recent studies found that GRK2 hemizygous mice showed increased NO bioavailability and that inducible genetic ablation of GRK2 reduced glucose tolerance and insulin resistance^16, 42^. Specifically, the present study provides evidence that acute normalization of glucose homeostasis increases Akt/eNOS signaling and NO bioavailability after GRK2-induced endothelial dysfunction. In this situation, strategies aimed at improving endothelial dysfunction in T2DM in general and restoring GRK2 expression in the liver in particular may provide the basis for the rational design of novel therapies for T2DM and its attendant diabetic vascular complications.

## Materials and Methods

All experiments were performed in accordance with the guidelines and approved by the Hoshi University Animal Care and Use Committee (accredited by the Ministry of Education, Culture, Sports, Science and Technology of Japan; approval number: 26-099, 27-062).

### Reagents

Clonidine chloride, insulin, nicotinamide, PE, STZ, and Mission Transduction particles Mm_GRK2_1630 (GRK2 siRNA) were purchased from Sigma Chemical Co. (St. Louis, MO, U.S.A.). GRK2-inhibitor was purchased from Calbiochem (San Diego, CA, U.S.A.). The company’s name for the GRK2-inhibitor (methyl [(5-nitro-2-furyl) vinyl] −2-furoate) is GRK2 inhibitor. Recombinant insulin glargine was purchased from Sanofi K.K. (Tokyo, Japan). Prostaglandin F_2α_ (PGF_2α_) was purchased from Fuji Pharma (Tokyo, Japan). ACh chloride was purchased from Daiichi-Sankyo Pharmaceuticals (Tokyo, Japan). SNP was obtained from Wako Pure Chemical Industries, Ltd. (Osaka, Japan). The concentrations of these drugs are expressed as final molar concentrations. Antibodies for Akt, Akt phosphorylated at Ser473 and Thr308, and eNOS phosphorylated at Ser1177 and Thr495, p44/42 MAPK (ERK1/2), phosphorylated p44/42 MAPK at Thr202/Tyr204, phosphorylated p38 MAPK at Thr180/Tyr182, p38 MAPK, AMPKα, AMPKβ2, and AMPKα phosphorylated at Thr172 were purchased from Cell Signaling Technology (Danvers, MA, U.S.A.); antibody for eNOS from BD Bioscience (San Jose, CA, U.S.A.); antibody for GRK2 from Santa Cruz Biotechnology (Santa Cruz, CA, U.S.A.); antibody for phosphorylated GRK2 at Ser670 from GeneTex Inc (Irvine, CA, U.S.A.); antibody for β-actin was from Sigma Chemical Co. Horseradish peroxidase-linked secondary anti-mouse and anti-rabbit antibody was purchased from Promega (Madison, WI, U.S.A.). All antibodies were diluted to appropriate concentrations.

### Animals

Four-week-old male Institute of Cancer Research (ICR) mice were obtained from Tokyo Animal Laboratories (Tokyo, Japan). Diabetic model mice (nicotinamide + STZ-induced diabetic mice) were induced by intraperitoneal injection of nicotinamide (1.5 g/kg body weight) in saline and were injected via the tail vein after 15 min with STZ (200 mg/kg body weight) dissolved in citrate buffer in 5 week-old ICR mice^22, 23^. Age-matched controls were injected with buffer alone. The experiments were performed 17–20 weeks after injection. Mice were divided randomly into three experimental groups: a control group: nondiabetic mice used as a control; DM group: nicotinamide (Nic) + STZ-induced diabetic; DM + GRK2 si group: Nic + STZ-induced diabetic mice were transfected with GRK2 siRNA.

Male *ob/ob* mice and age-matched Lean (control) C57BL/6J mice (27–32 weeks old) were obtained at the age of 5 weeks (Sankyo Labo Service Corporation, INC., Tokyo, Japan). Lean mice were transfected with control siRNA (lean-cont siRNA). *Ob/ob* mice were divided randomly into two experimental groups; the *ob/ob*-cont siRNA group: *ob/ob* mice which were transfected with control siRNA; and the *ob/ob*-GRK2 siRNA group: *ob/ob* mice which were transfected with GRK2 siRNA. Water and food were given ad libitum in a controlled environment (room temperature: 21–22 °C, humidity: 50 ± 5%). This study was performed in accordance with the guide issued by the Hoshi University Animal Care and Use Committee (accredited by the Ministry of Education, Culture, Sports, Science and Technology of Japan).

### Cell culture

Human umbilical vein endothelial cells (HUVECs) were purchased from Kurabo (Osaka, Japan). The cells were subcultured in HuMedia-EG2 growth medium (Kurabo) and grown at 37 °C in a humidified atmosphere in a 5% CO_2_ incubator. Cells from passages 4–7 were used in all experiments and in 12-well plates at a density of 3 × 10^5^ cells/wells. To mimic a diabetic environment (hyperglycemic and hyperinsulinemic conditions), HUVECs were exposed to low or high (5 or 22 × 10^−3^ mol/L) glucose in the presence or absence of insulin (10^−7^ mol/L) for 72 h. Briefly, the experimental groups were divided into a normal control group (L/L: HUVECs were incubated in low- glucose medium for 72 h and, over the next 0–24 h, replaced low glucose medium), a quasi-T2DM group (H/H: HUVECs were incubated in high glucose/high insulin medium for 72 h and, over the next 0–24 h, replaced high glucose/high insulin medium), and a cure group (H/L: HUVECs were incubated in high glucose/high insulin medium for 72 h, and over the next 0–24 h, replaced low glucose medium). Finally, the cells of the three groups were either stimulated with insulin (10^−6^ mol/L) or not stimulated for 20 min.

### *In vivo* delivery of siRNA using hydrodynamic injection

siRNA sequences matching GRK2 and a scrambled siRNA (used as a control) were purchased commercially from Sigma. These siRNAs were independently verified for efficacy and specificity by Sigma. To transfect siRNA into mouse livers, we used hydrodynamic injection as described previously^25, 27, 43^. The siRNA in a hydrodynamic delivery solution (Mirus Bio, WI, U.S.A.) was injected into the tail vein over 5 s in accordance with the manufacturer’s instructions. For GRK2 siRNA and control siRNA, 1–2 mg/kg of siRNA was injected. Liver and aorta tissues were removed from each mouse 24 h after hydrodynamic injection. In some experiments, insulin (1 U/kg) was administered intraperitoneally and liver tissue was removed after 15 min.

### Measurement of plasma concentrations of glucose and insulin

Plasma samples were collected from the abdominal aorta of each mouse at the indicated times after hydrodynamic injection of siRNA. The concentration of glucose in the plasma was measured with a commercially available enzyme kit (Wako Chemical Company, Osaka, Japan) and the concentration of insulin in the plasma was measured using an enzyme-linked immunosorbent assay (ELISA) kit (Shibayagi, Gunma, Japan) as reported previously^22, 23, 31^.

### Intraperitoneal glucose and insulin tolerance tests

An IGTT and IITT were performed as previously described^22, 23, 31^. Briefly, after 24 h of siRNA hydrodynamic injection, glucose (2 mg/kg body weight as a 30% solution) was given intraperitoneally to 18-h-fasting mice. At 24 h after siRNA hydrodynamic injection, human insulin (0.75 U/kg) was administered to conscious non-fasting mice via the intraperitoneal route.

### Measurement of isometric force

Vascular reactivity of mouse aorta was studied as previously described^10, 22–24, 31^. Isolated thoracic aorta was cleaned of connective tissue and cut into rings (2 mm) in Krebs-Henseleit solution (KHS; composition in ×10^−3^ mol/L: NaCl 118.0, KCl 4.7, NaHCO_3_ 25.0, CaCl_2_ 1.8, NaH_2_PO_4_ 1.2, MgSO_4_ 1.2, glucose 11.0, at 37 °C). The rings were placed in organ baths containing oxygenated (95% O_2_ –, 5% CO_2_) KHS. Following equilibration for 45 min under an optimal resting tension of 1.5 g, the rings were contracted with KCl (80 × 10^−3^ mol/L) to check their reactivity. After washout and a 45-min equilibration period, the rings were contracted again with PGF_2α_ (10^−6^–3 × 10^−6^ mol/L) to approximately 80% of the maximal contraction and reached a plateau level, before construction of a concentration–relaxation curve with clonidine (10^−9^–10^−6^ mol/L), insulin (10^−9^–3 × 10^−6^ mol/L), ACh (10^−9^–10^−5^ mol/L), or SNP (10^−10^–10^−5^ mol/L). In another set of experiments, concentration-response curves to PE (10^−9^–10^−4^ mol/L) were performed. Vascular constriction responses were expressed as a percentage of the tone generated by KCl. KCl-induced responses were similar in the aortas from all of the groups of mice. Vascular relaxation responses were expressed as a percentage of the tone generated by PGF_2α_.

### Measurement of NO production

NOx (nitrite + nitrate) was measured as an index of NO by high-performance liquid chromatography (ENO20, Eicom, Kyoto, Japan) in the effluent from each aorta and the medium from each HUVEC, as previously described^10, 22–24^.

### Measurement of protein expression

Each organ and also the HUVECs were homogenized, dissolved in RIPA buffer (Thermo Scientific, Rockford, IN, U.S.A.) containing protease and phosphatase inhibitor cocktail (Roche Diagnostics, Indianapolis, IN, U.S.A.), and the lysates were used for western blot analysis as previously described^10, 22–24, 31^. Protein concentrations were measured with a BCA protein assay kit (Pierce, Rockford, IL, U.S.A.). Equal amounts of protein were loaded into lanes of 10% sodium dodecyl sulfate polyacrylamide gel electrophoresis gels. The gels were electrophoresed and the protein was transferred to a polyvinyl difluoride membrane. The membrane was then blocked and probed with primary antibodies overnight at 4 °C. The following primary antibodies were used: phosphorylated GRK2 Ser 670 (1:1,000); GRK2 (1:200); phosphorylated Akt Ser473 (1:1,000); phosphorylated Akt Thr308 (1:1,000); Akt (1:1,000); phosphorylated eNOS Ser1177 (1:1,000); phosphorylated eNOS (Thr495) (1:1,000); eNOS (1:1,000); phosphorylated AMPK Thr172 (1:1,000); AMPKα (1:1,000), AMPKβ (1:1,000); phosphorylated p44/42 MAPK Thr202/Tyr204 (ERK1/2; 1:2,000); ERK1/2 (1:2,000); phosphorylated p38 MAPK Thr180/Tyr182 (1:2,000); p38 MAPK (1:2,000); β-actin (1:5,000). Immunoblots were next processed with the appropriate secondary antibodies (1:10,000) for 20 min at 37 °C. Immunoblots were then probed with a SuperSignal (Thermo Scientific) to visualize signal with Light-capture (ATTO, Tokyo, Japan). The optical densities were quantified with CS Analyzer 3.0 software (ATTO). To normalize the data, we used β-actin as a housekeeping protein. Ratios were calculated for the optical densities of phosphorylated Akt, eNOS or AMPK to those of the corresponding total protein bands.

### Statistical analysis

All statistical analyses were performed with Graph Pad Prism 6.0 (Graph Pad Software Inc., San Diego, CA, U.S.A.). Values are presented as means ± SE. Statistical differences were assessed by Bonferroni’s test for multiple comparisons after one- or two-way analysis of variance (ANOVA), with *p* < 0.05 being considered significant. Statistical comparisons between concentration–response curves were made using repeated measures two-factor ANOVA test, with *post hoc* correction for multiple comparisons by Bonferroni’s test, with *p* < 0.05 considered significant. GRK2 activity was calculated as be the reciprocal of phosphorylated GRK2 expression.

## Electronic supplementary material

Supplemental data
