# Supplementary material for: Suppression of GRK2 expression reduces endothelial dysfunction by restoring glucose homeostasis
Source: Sci Rep. 2017 Aug 16;7:8436. doi: 10.1038/s41598-017-08998-5 (PMC5559446; doi:10.1038/s41598-017-08998-5)
Supplement: Supplementary file 1 — Supplemental data [file 41598_2017_8998_MOESM1_ESM.doc]

**Supplemental data**

**Suppression of GRK2 expression reduces endothelial dysfunction by restoring glucose homeostasis**

Kumiko Taguchi, Mari Hida, Mami Hasegawa, Haruka Narimatsu, Takayuki Matsumoto, Tsuneo Kobayashi*

*Department of Physiology and Morphology, Institute of Medicinal Chemistry,*

*Hoshi University, Shinagawa-ku, Tokyo 142-8501, Japan*

*Corresponding author. Tel. and Fax: +81-3-5498-5849

E-mail: [tkoba@hoshi.ac.jp](mailto:tkoba@hoshi.ac.jp)

**
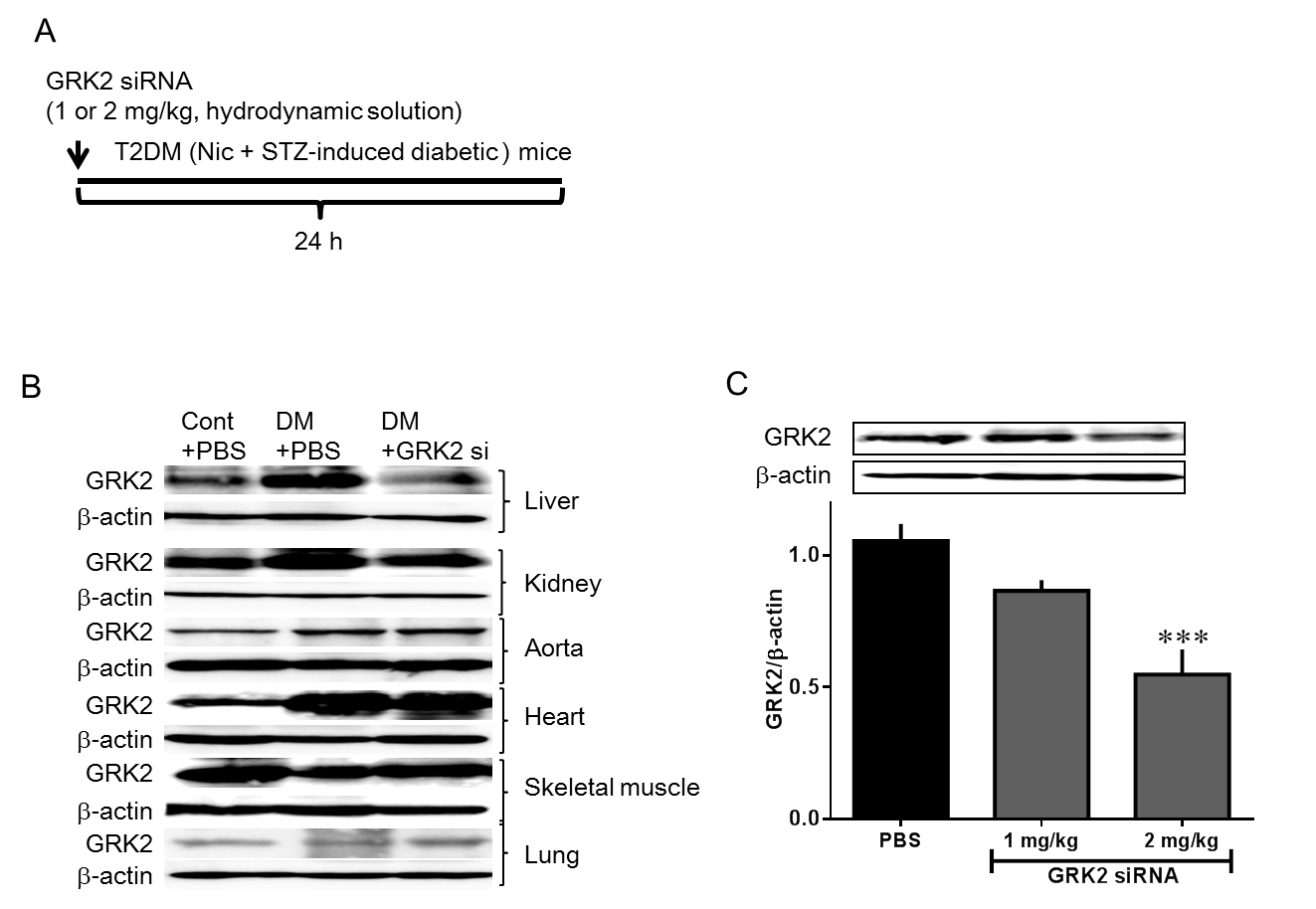
**

**Supplemental Fig. 1.** (A) Experimental protocol for the T2DM model (nicotinamide + streptozotocin [STZ]-induced diabetic) mice. T2DM mice were treated with GRK2 siRNA using hydrodynamic delivery as indicated (1 or 2 mg/kg; intravenously). (B) Representative western blot analysis of GRK2 and β-actin in liver, kidney, aorta, heart, skeletal muscle, and lung from control, diabetes mellitus (DM), or GRK2 siRNA-transfected DM mice. (C) T2DM model mice were injected with a hydrodynamic solution with 1 or 2 mg/kg GRK2 siRNA. The livers were isolated from T2DM model mice or GRK2 siRNA-transfected T2DM mice for western blot analysis. Values are mean ± SE; *n* = 6. ^***^*p <* 0.001 vs. control (injected with phosphate-buffered saline).


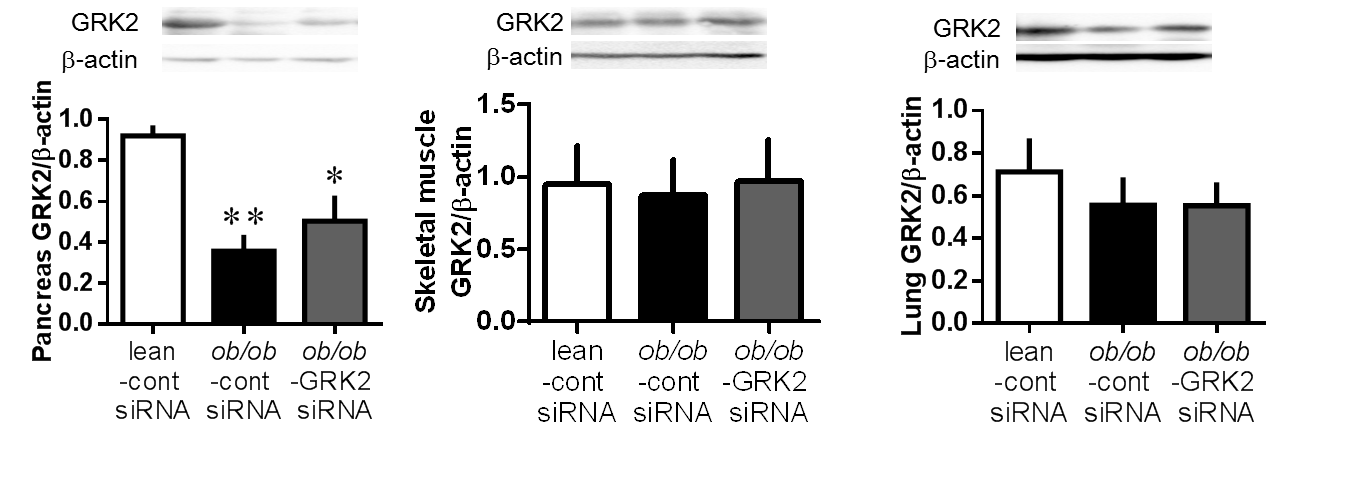


**Supplemental Fig. 2.** GRK2 is reduced by GRK2 siRNA delivery in liver. *ob/ob* mice or lean (control) mice were treated with GRK2 siRNA or control siRNA by hydrodynamic delivery as indicated (intravenously. 2 mg/kg). Western blot analysis of GRK2 in the pancreas, skeletal muscle, and lung. Values are mean ± SE; *n* = 6. ^*^*p <* 0.05, ^**^*p <* 0.01 vs. control siRNA-transfected lean mice (lean-cont siRNA).

**
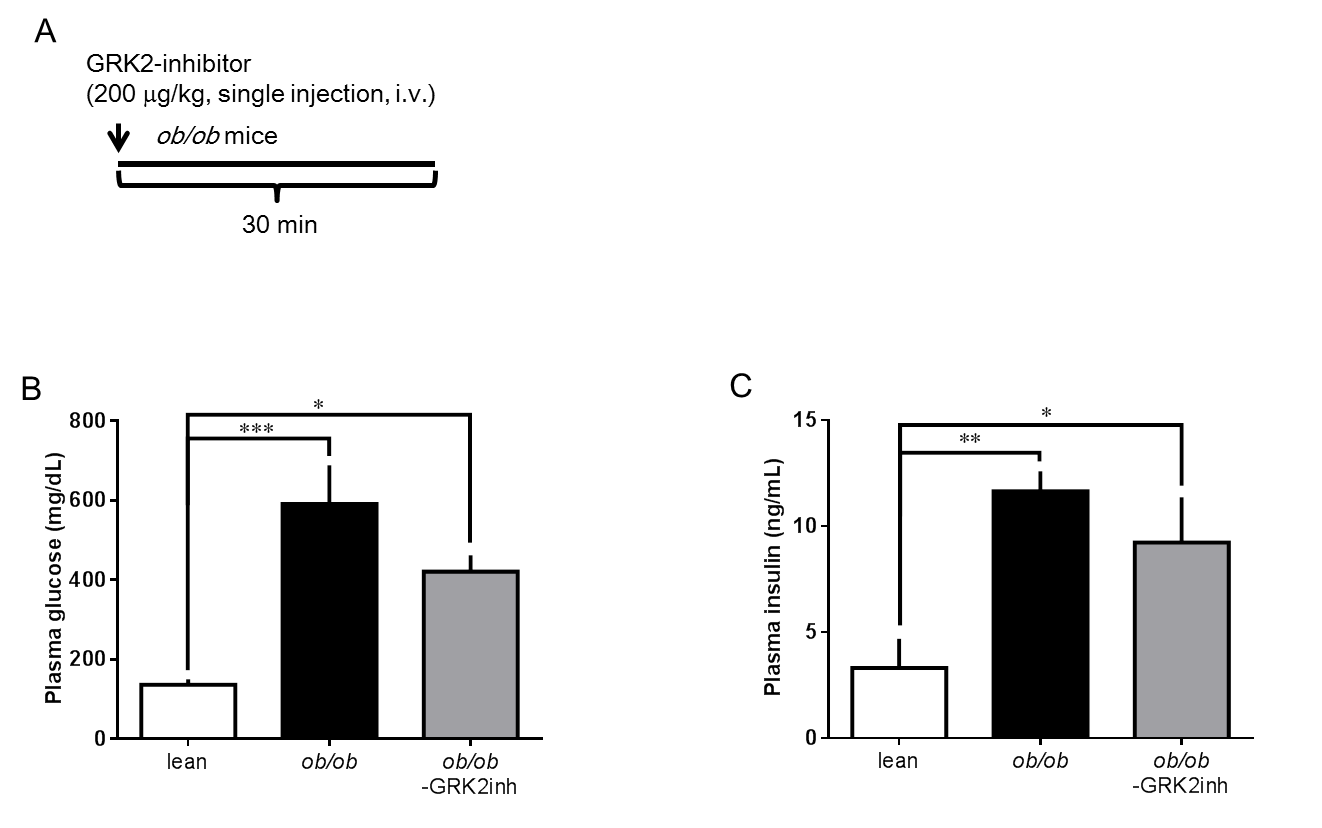
**

**Supplemental Fig. 3.** (A) Experimental protocol for *ob/ob* mice. *ob/ob* mice were administered a single dose of GRK2 inhibitor (200 μg/kg; intravenously). (B, C) Non-fasting plasma levels of glucose (B) and insulin (C) were determined in lean, *ob/ob* and GRK2 inhibitor-injected *ob/ob* mice (*ob/ob*-GRK2 inh). Values are mean ± SE; *n* = 6. ^*^*p <* 0.05, ^**^*p <* 0.01, ^***^*p <* 0.001 vs. lean mice.

**
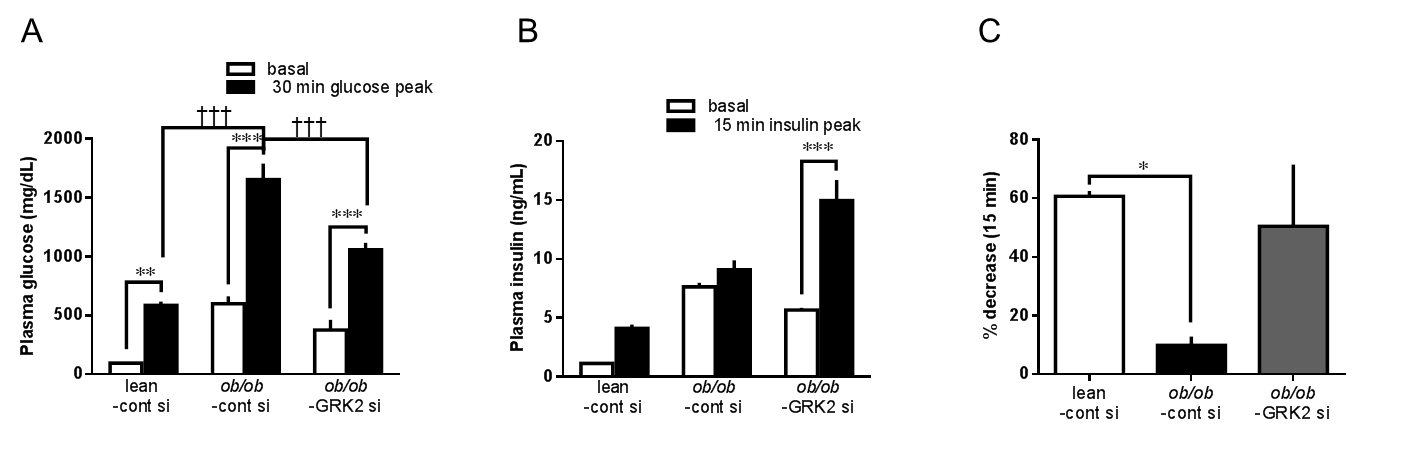
**

**Supplemental Fig. 4.** Suppression of hepatic GRK2 restores glucose homeostasis and insulin sensitivity in *ob/ob* mice. Plasma levels of glucose and insulin were determined during a IGTT (glucose 2.0 g/kg intraperitoneally [i.p.]) performed 24 h after GRK2 siRNA injection. Values of plasma glucose at baseline and 30 min after glucose loading (A). Plasma insulin levels at baseline and 15 min after glucose loading (insulin peak) (B). Plasma levels of glucose was determined during an IITT (insulin 0.75 U/kg i.p.) performed after 24 h of GRK2 siRNA injection. Percent decrease in plasma glucose values with respect to the basal levels and those at 15 min after insulin administration (C). Values are mean ± SE; *n* = 6. ^*^*p <* 0.05 vs. control siRNA-transfected lean mice (lean-cont si); ^**^*p <* 0.01, ^***^*p <* 0.001 vs. basal. ^†††^*p <* 0.001 vs. control siRNA-transfected *ob/ob* mice (*ob/ob*-cont si).

**
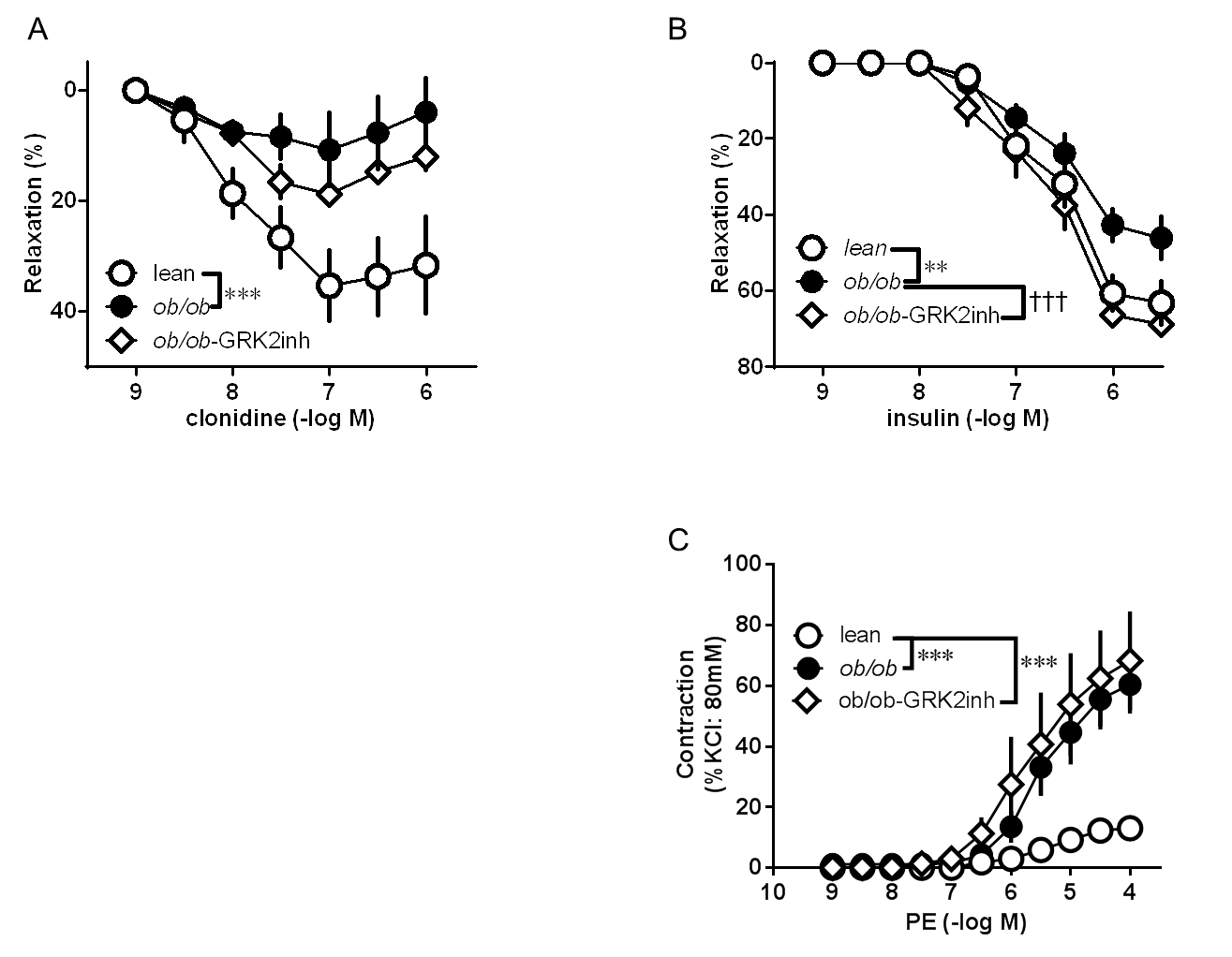
**

**Supplemental Fig. 5.** Concentration–response curves for clonidine (A), insulin (B), and PE (C) in aortas from lean, *ob/ob,* and GRK2-inhibitor injected-*ob/ob* mice (*ob/ob*-GRK2-inh). *Ob/ob* mice were treated with phosphate-buffered saline or GRK2 inhibitor using the dosing regimen outlined in Supplemental Fig. 3. Values are mean ± SE; *n* = 4. ^**^ *p* < 0.01, ^***^*p <* 0.001 vs. lean; ^†††^*p <* 0.001 vs. *ob/ob* mice.

**
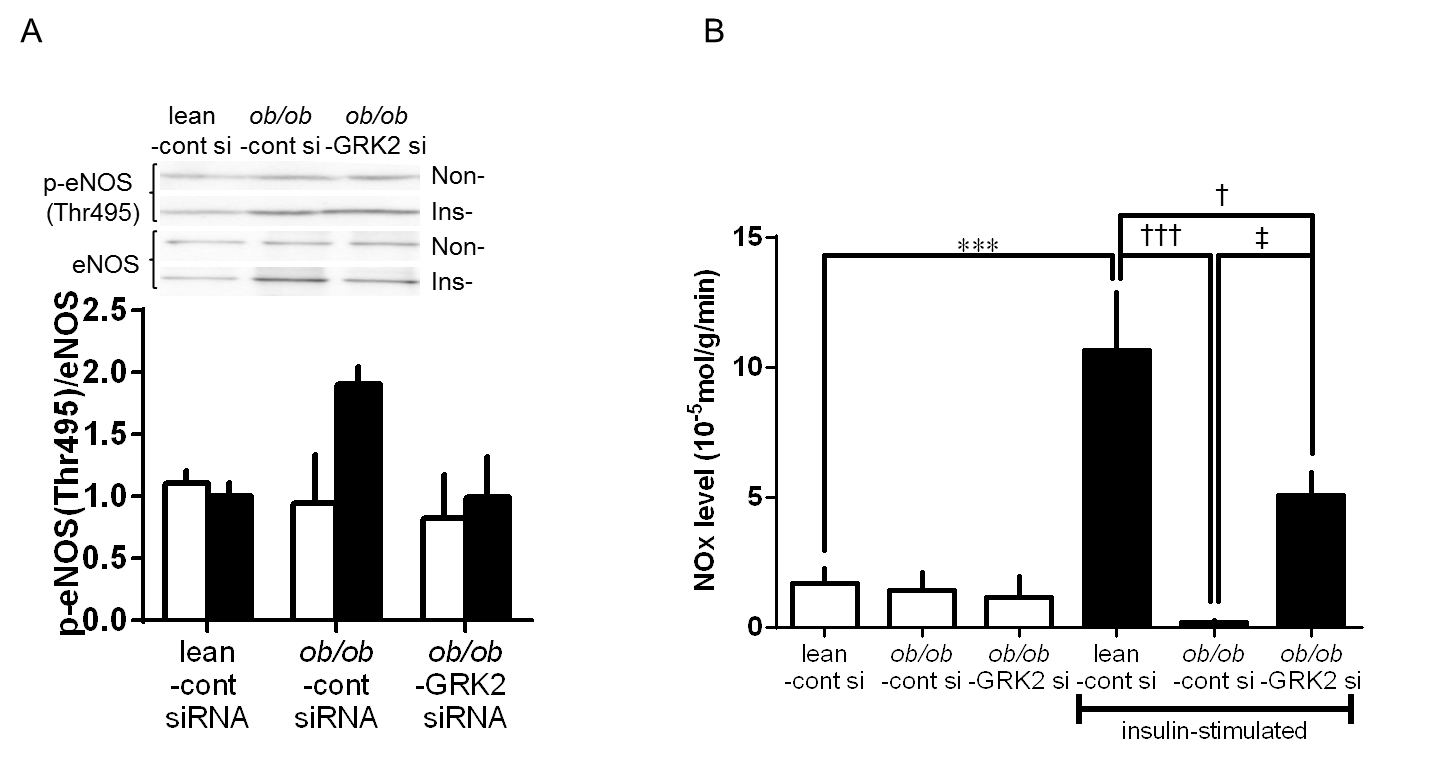
**

**Supplemental Fig. 6.** Effect of suppression of hepatic GRK2 on insulin signaling in *ob/ob* mice. (A) Results are expressed as ratio of eNOS phosphorylation (at Thr495) to total eNOS (p-eNOS [Thr495]). (B) NO production in aortas isolated from control siRNA-transfected lean (lean-cont si), control siRNA-transfected *ob/ob* (*ob/ob*-cont si), and GRK2 siRNA-transfected *ob/ob* mice (*ob/ob*-GRK2 si). Mice were treated with control siRNA or GRK2 siRNA using the dosing regimen outlined in Fig. 1 and stimulated with insulin (10^-6^ mol/L; 20 min) after aorta isolation. Values are mean ± SE; *n* = 6-8. ^***^*p* < 0.001 vs. lean-cont si (non-stimulated); ^†^*p* < 0.05, ^†††^*p* < 0.001 vs. lean-cont si (insulin-stimulated); ^‡^*p* < 0.05 vs. *ob/ob*-cont si (insulin-stimulated).

**
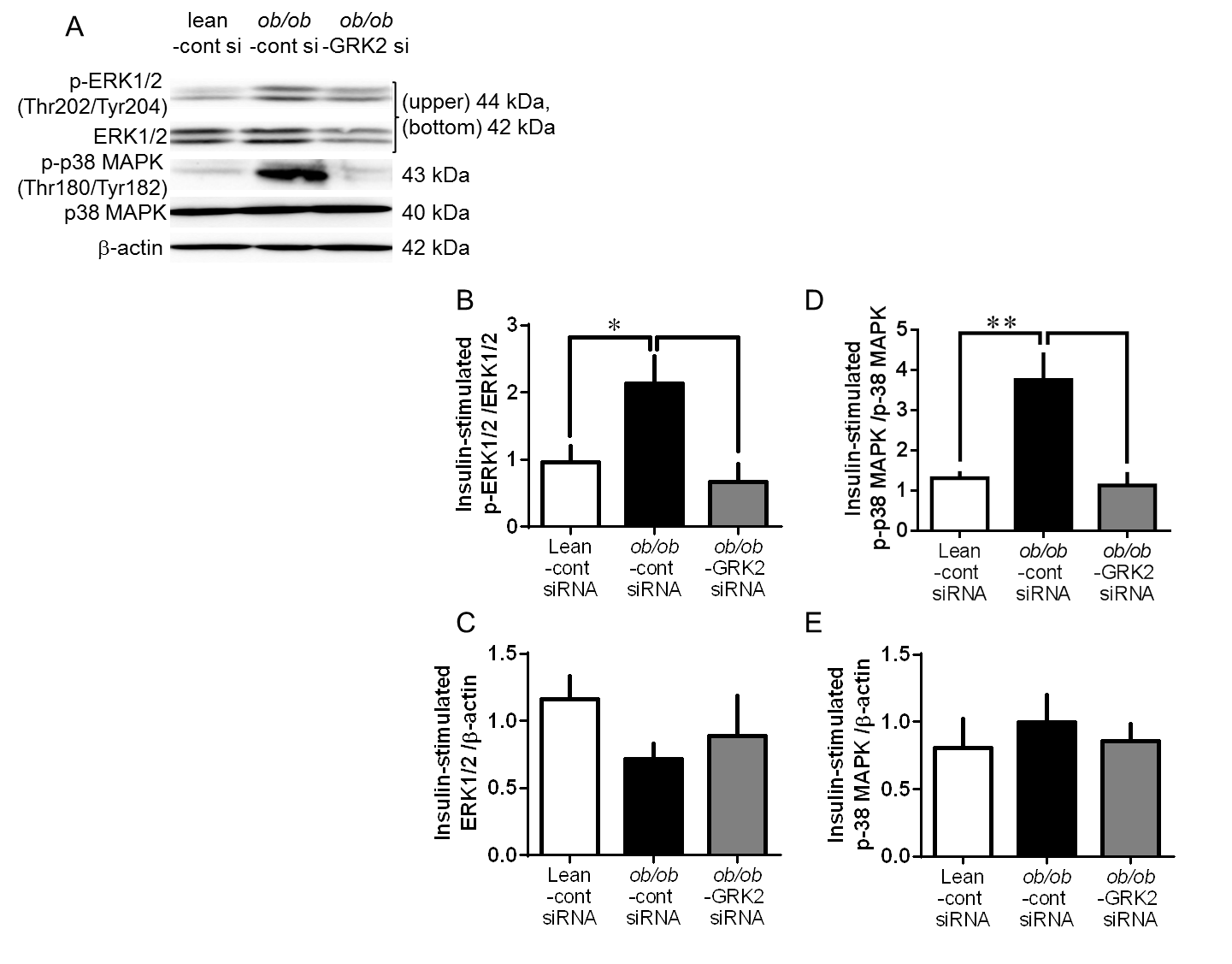
**

**Supplemental Fig. 7.** (A) Representative western blot analysis of phospho-ERK1/2, total ERK1/2, phospho-p38 MAPK, total p38 MAPK, and β-actin in aortas from control siRNA-transfected lean (lean-cont si), control siRNA-transfected *ob/ob* (*ob/ob*-cont si), or GRK2 siRNA-transfected *ob/ob* (*ob/ob*-GRK2 si) mice under insulin stimulation (10^-6^ mol/L; 20 min). (B-E) Results are expressed as the ratio of ERK1/2 phosphorylation (p-ERK1/2 (Thr202/Tyr204) to total ERK1/2 (B), ratio of ERK1/2 to β-actin (C), p38 MAPK phosphorylation (p-p38 MAPK [Thr180/Tyr182]) to total p38 MAPK (D), and ratio of p38 MAPK to β-actin (E). Values are mean ± SE; *n* = 6. ^*^ *p* < 0.05, ^**^*p <* 0.01 vs. lean-cont si; ^†^*p* < 0.05, ^††^*p <* 0.01 vs. *ob/ob*-cont si.


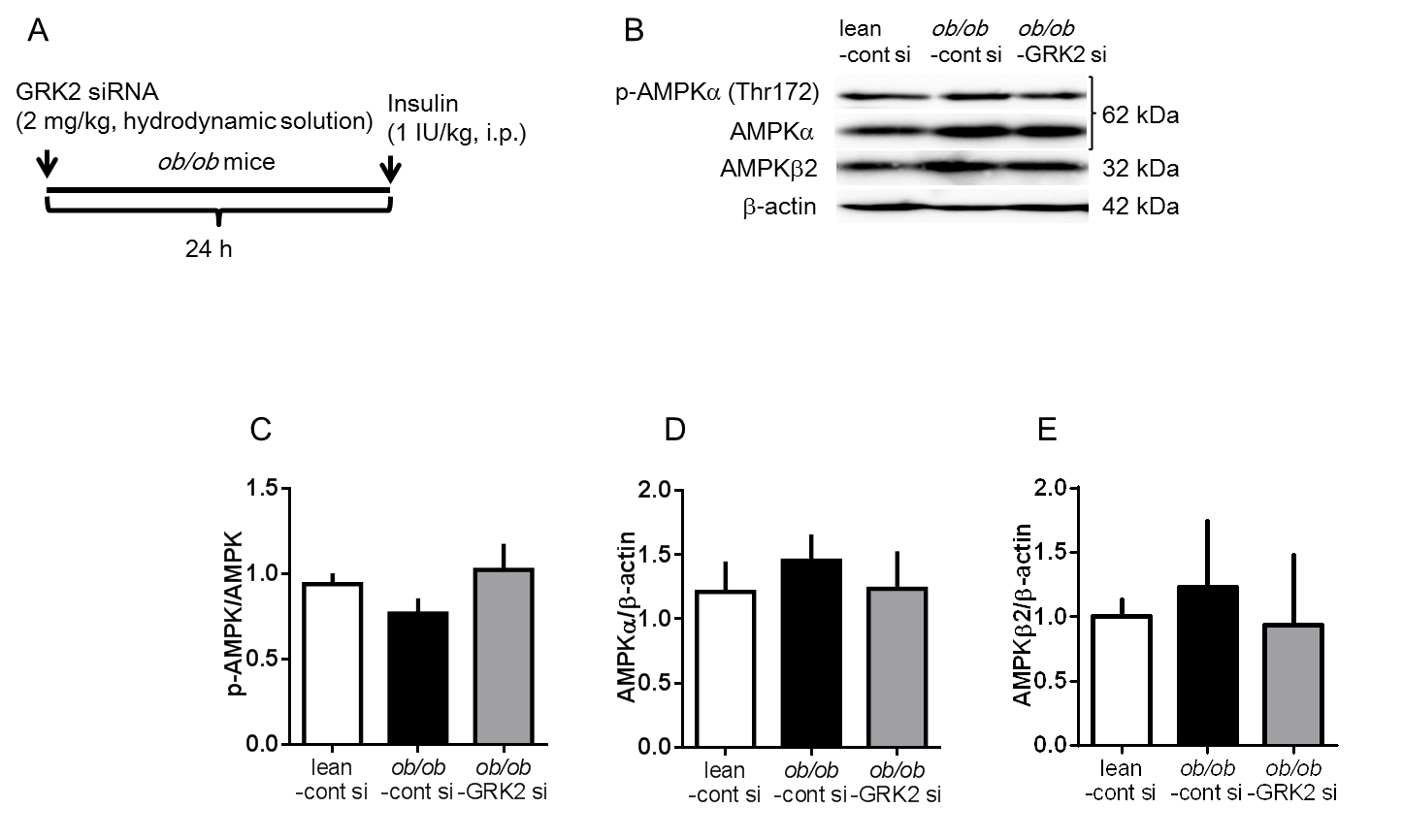


**Supplemental Fig. 8.** (A) Experimental protocol for *ob/ob* mice. *ob/ob* mice were treated with GRK2 siRNA by hydrodynamic delivery as indicated (2 mg/kg; intravenously). Insulin (1 IU/kg) was then injected intraperitoneally 24 h after treatment with GRK2 siRNA. (B) Representative western blot analysis of phospho-AMPKα, AMPKα, AMPKβ2, and β-actin in liver tissue from control siRNA-transfected lean (lean-cont si), control siRNA-transfected *ob/ob* (*ob/ob*-cont si), or GRK2 siRNA-transfected *ob/ob* (*ob/ob*-GRK2 si) mice. (C-E) Results are expressed as ratio of AMPKα phosphorylation (p-AMPK [Thr172] to total AMPKα (C) or ratio of AMPKα to β-actin (D), or AMPKβ2 to β-actin (E). Values are mean ± SE; *n* = 6.

**
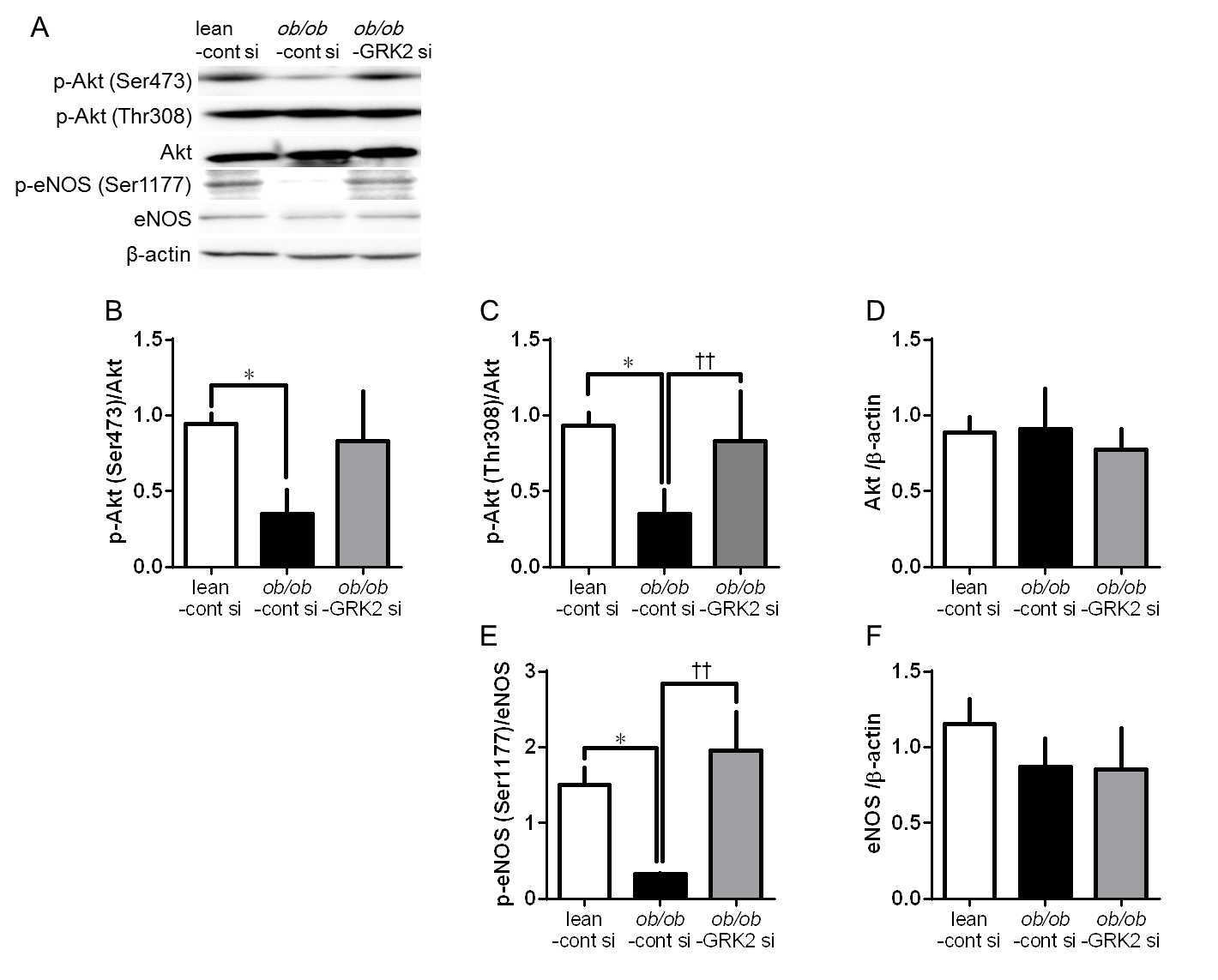
**

**Supplemental Fig. 9.** Western blots of liver from mice described in Supplemental Figure 6 were analyzed with antibodies against Akt phosphorylation (Ser473 and Thr308), total Akt, eNOS phosphorylation (Ser1177), and total eNOS. (A) Representative western blot analysis of phospho-Akt (Ser473 and Thr308), Akt, phosphor-eNOS (Ser1177), eNOS, and β-actin in the liver of from control siRNA-transfected lean (lean-cont si), control siRNA-transfected *ob/ob* (*ob/ob*-cont si), or GRK2 siRNA-transfected *ob/ob* (*ob/ob*-GRK2 si) mice. (B-F) Results are expressed as ratio of Akt phosphorylation (p-Akt [Ser473]) to total Akt (B), ratio of Akt phosphorylation (p-Akt [Thr308]) to total Akt (C), ratio of Akt to β-actin (D), ratio of eNOS phosphorylation (p-eNOS [Ser1177] to total eNOS (E), and ratio of eNOS to β-actin (F). Values are mean ± SE; *n* = 6. ^*^ *p* < 0.05 vs. lean-cont si; ^††^*p <* 0.01 vs. *ob/ob*-cont si.

**
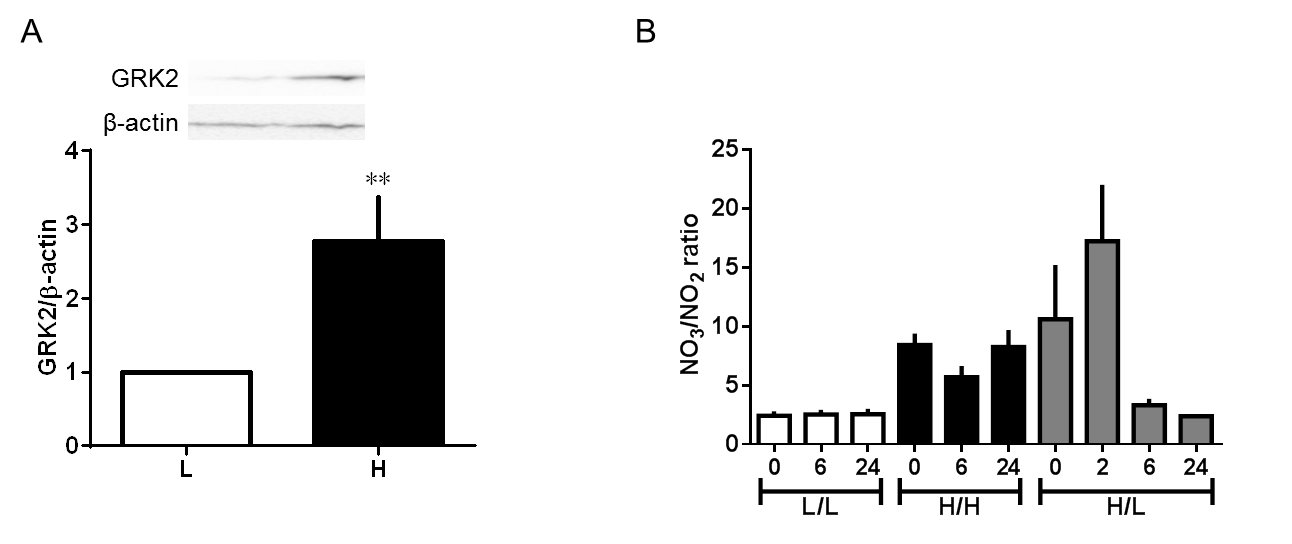
**

**Supplemental Fig. 10.** HUVECs were incubated with LG (5 mmol/L) (L) or HG (22 mmol/L) /HI (1 μmol/L) (H) for 72 h as described in Figure 6. (A) Western blot analysis of GRK2 expression in HUVECs. (B) Ratio of NO_3_ to NO_2_ from HUVECs or GRK2-induced HUVECs was measured under insulin stimulation or non-stimulation. Data are presented as insulin-stimulated NO production after deduction of non-stimulated NO production, and the ratio of NO_3_ to NO_2_ values was calculated. Three groups of HUVECs are indicated as follows: for L/L, HUVECs were incubated in LG medium for 72 h and over the next 0-24 h LG medium was replaced (control); for H/H, HUVECs were incubated in HG/HI medium for 72 h and over the next 0-24 h HG/HI medium was replaced (quasi-diabetic state); and H/L, HUVECs were incubated in HG/HI medium for 72 h and over the next 0-24 h LG medium was replaced. Values are mean ± SE; *n* = 6. ^**^*p* < 0.01 vs. L.

**
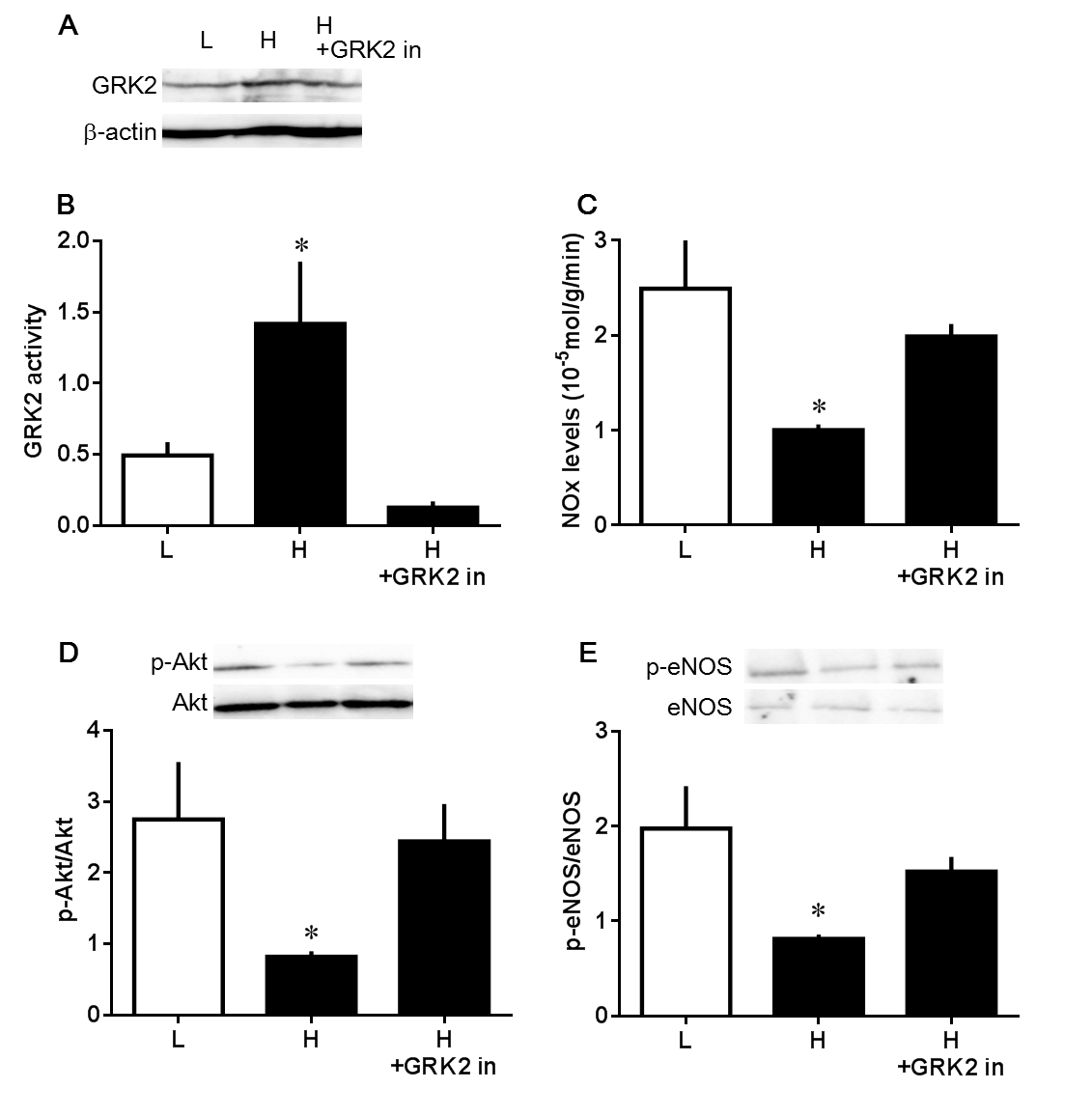
**

**Supplemental Fig. 11.** HUVECs were incubated with LG (5 mmol/L) (L) or HG (22 mmol/L)/HI (1 μmol/L) (H) for 72 h as described in Figure 6. Three groups of HUVECs are incubated as follows: for L, HUVECs were incubated in L medium for 72 h (control), for H, HUVECs were incubated in HG/HI medium for 72 h (quasi-diabetic state), and H + GRK2 in, HUVECs were incubated in HG/HI medium and GRK2 inhibitor (1 μmol/L) for 72 h. (A) We confirmed whether the GRK2 expression was induced under the H and/or GRK2 inhibitor condition in HUVECs. (B) Levels of GRK2 activity in HUVECs. (C) NOx production under clonidine stimulation (1 μmol/L; 15 min). (D, E) Top: representative Western blots for Akt phosphorylation at Ser473 (D) and eNOS phosphorylation at Ser1177 (E) in clonidine-stimulated HUVECs. Bottom: bands were quantified. Phosphorylated proteins were normalized to total proteins. Values are mean ± SE; *n* = 4. *p < 0.05 vs. L.

**Supplemental Fig. 12.**

**
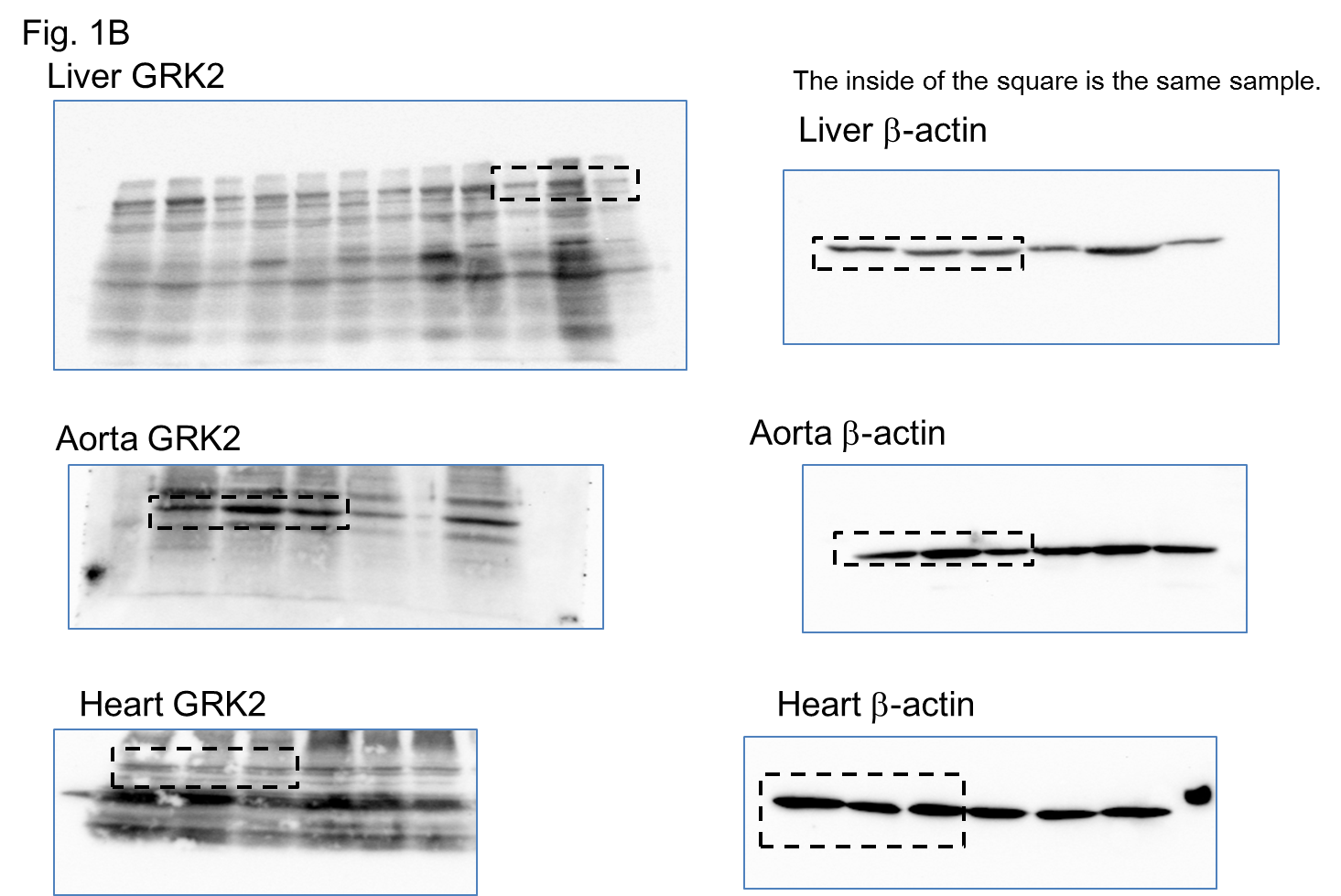
**

**
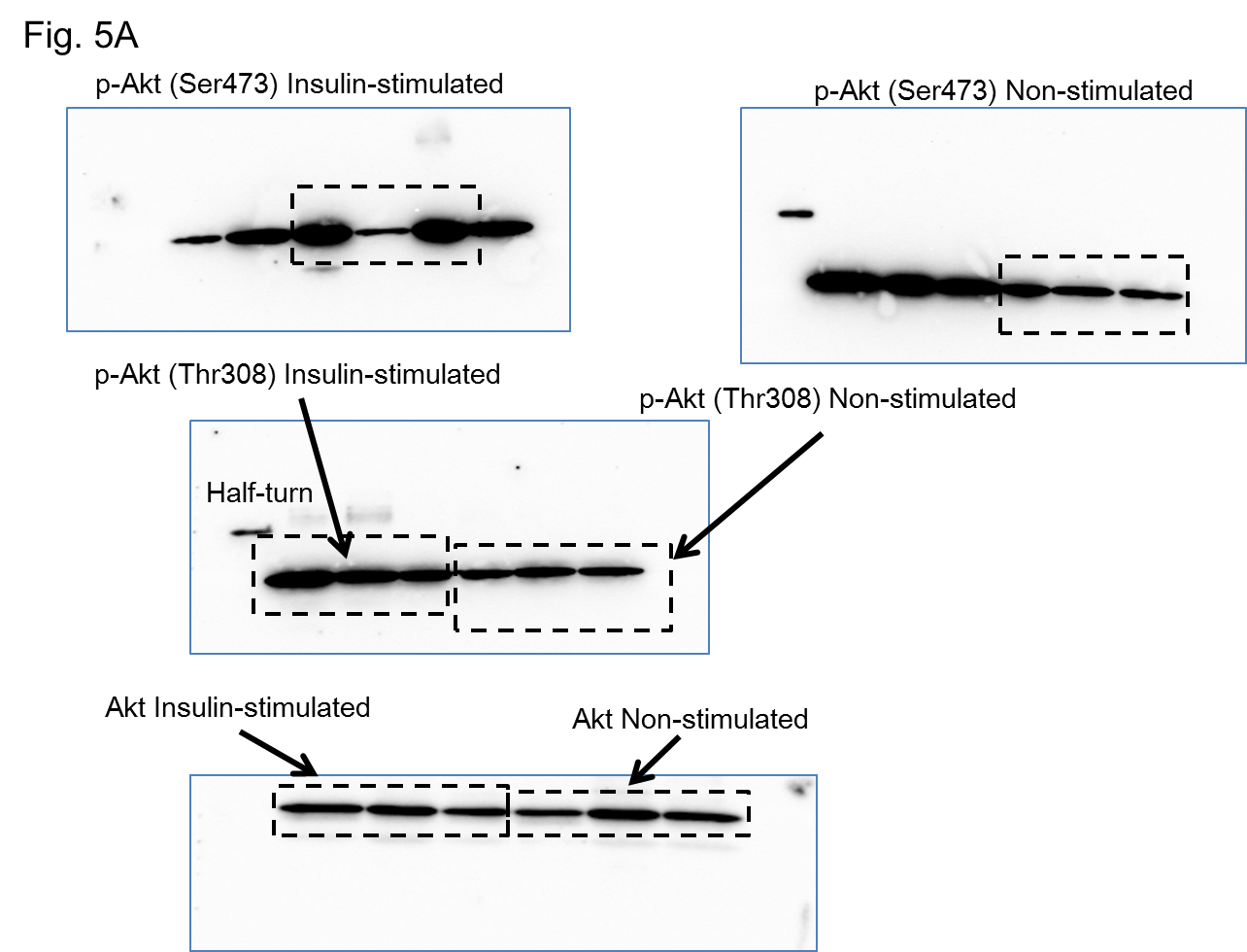
**

**
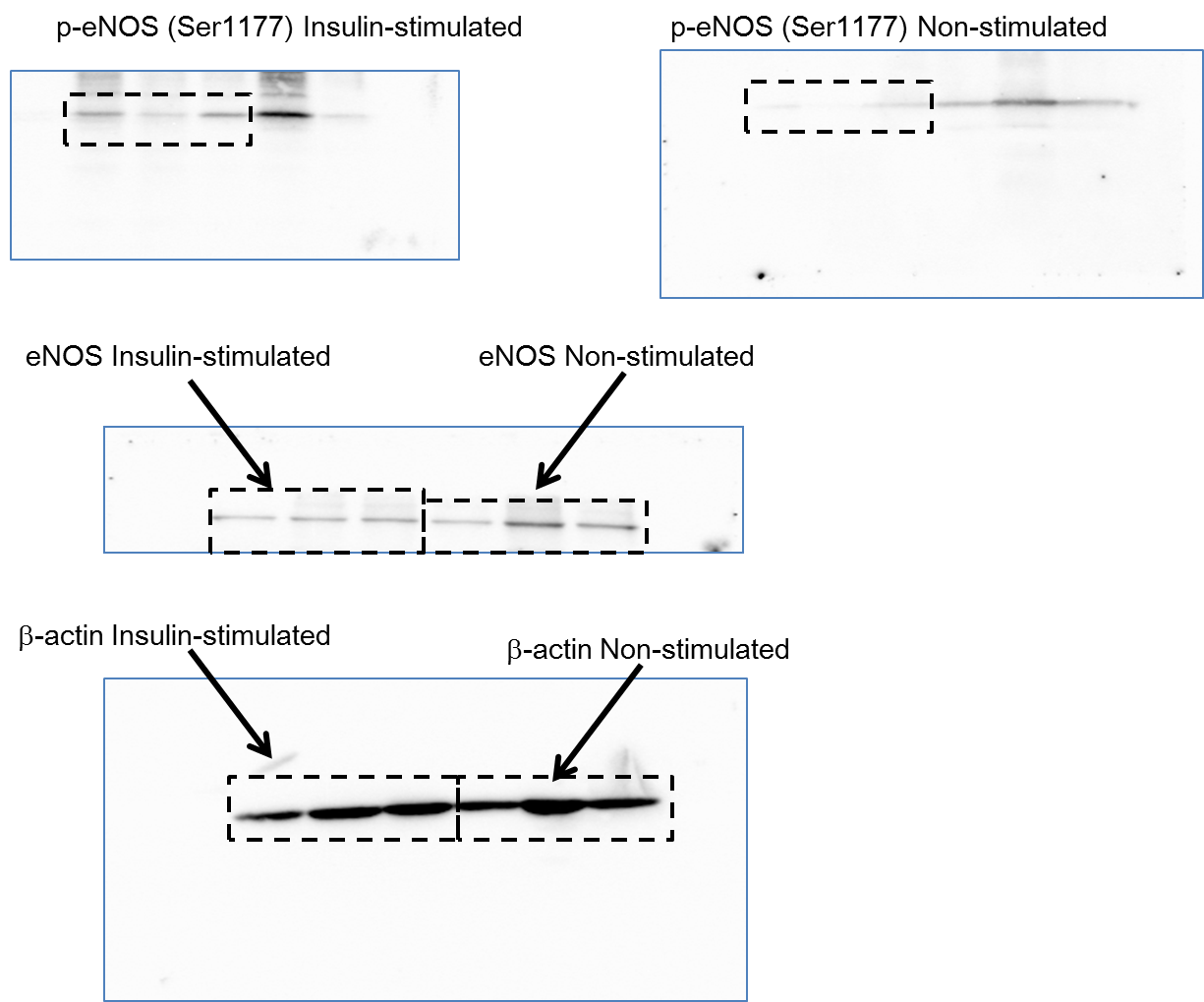
**

**
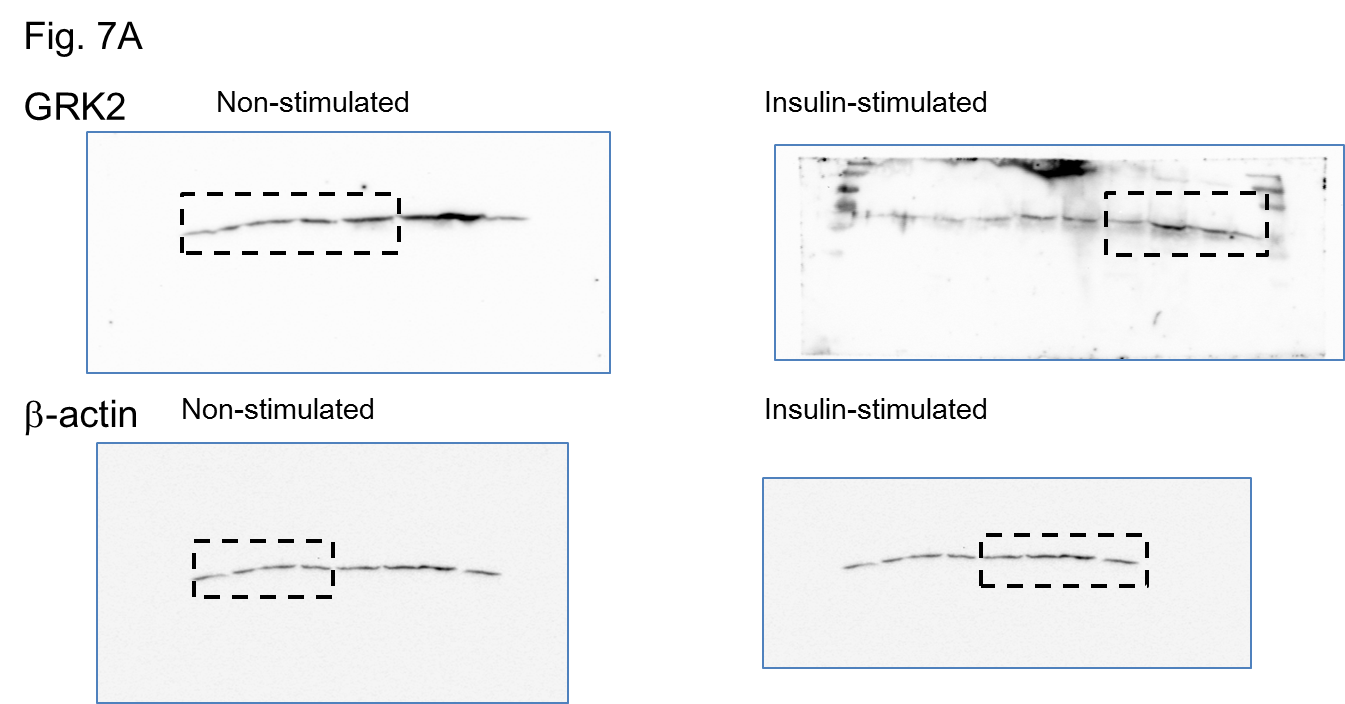
**

**
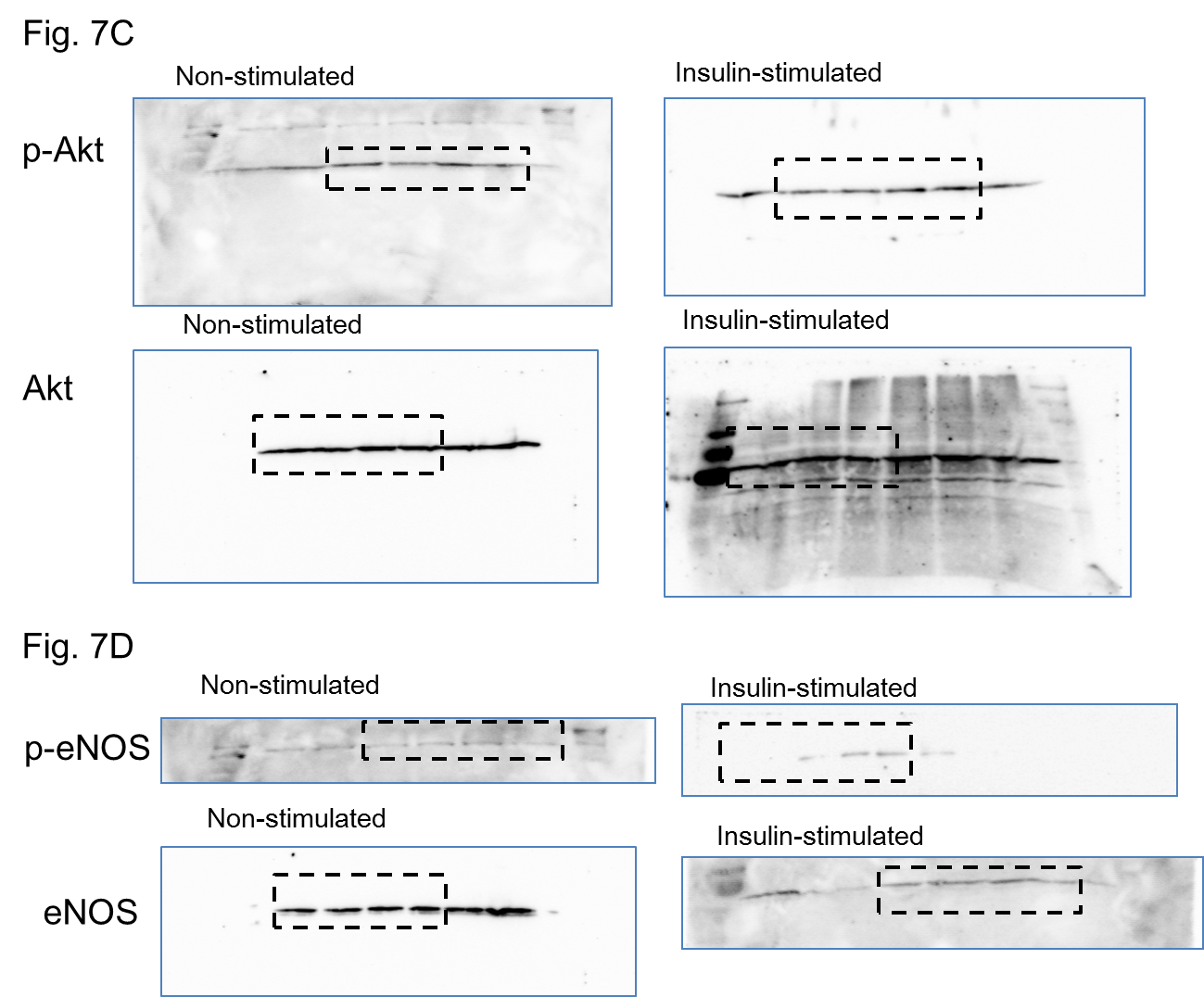
**

**
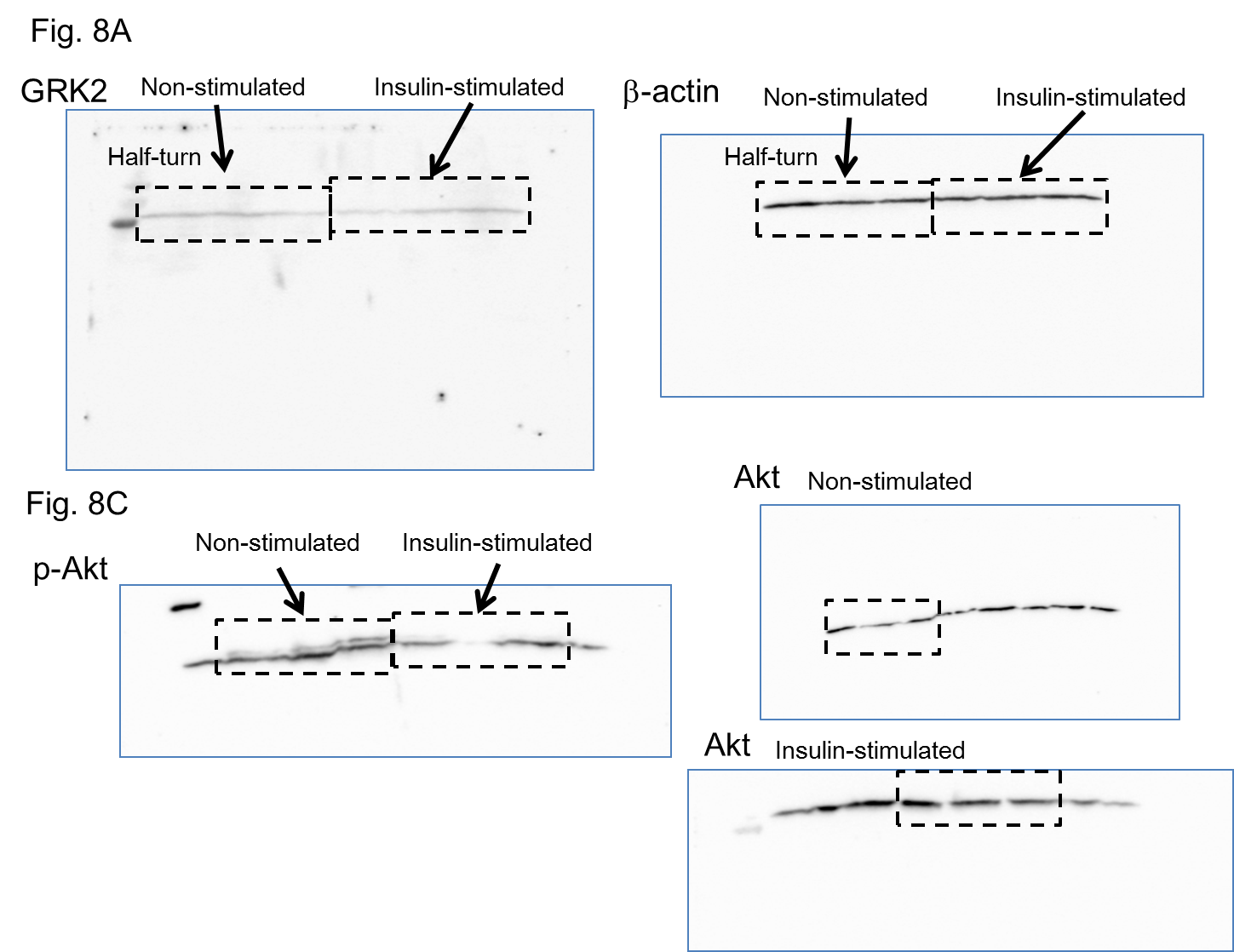
**

**
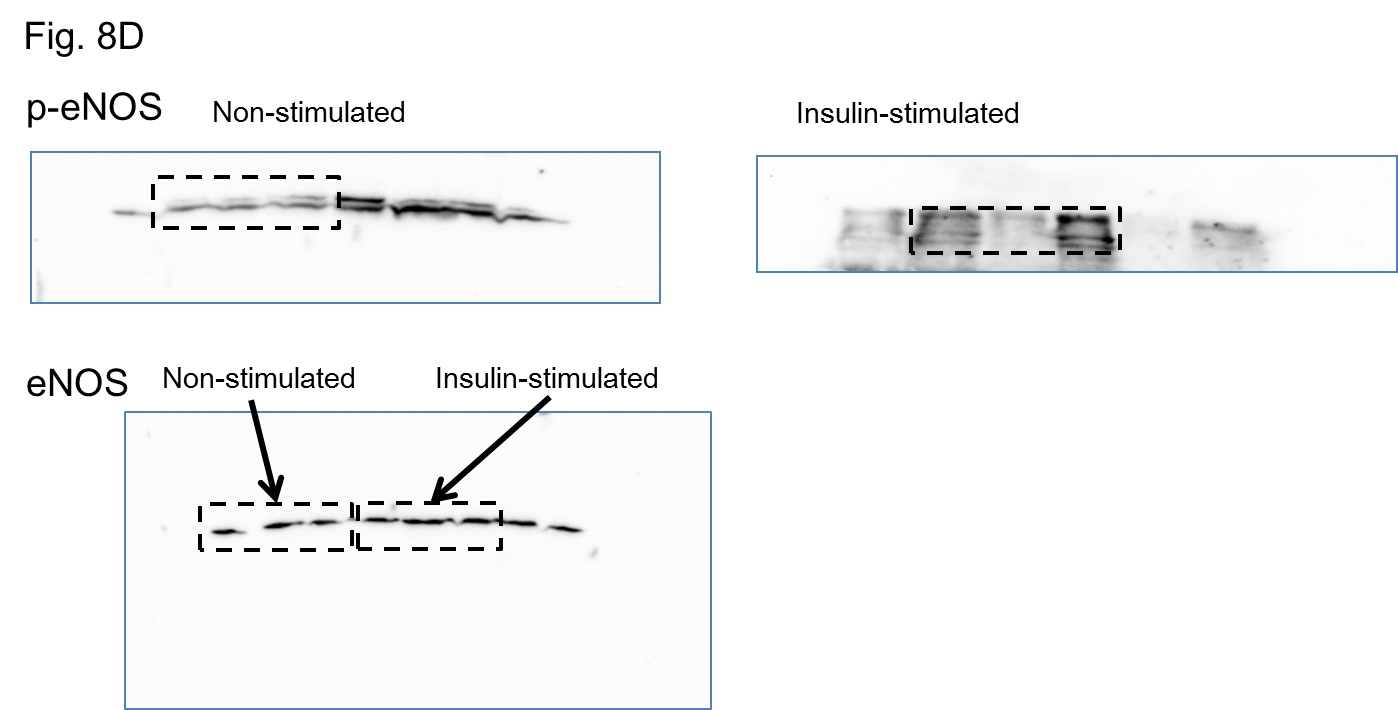
**
